# Spatiotemporally controlled drug delivery via photothermally driven conformational change of self-integrated plasmonic hybrid nanogels

**DOI:** 10.1186/s12951-023-01935-x

**Published:** 2023-06-14

**Authors:** Seungki Lee, Subeen Kim, Doyun Kim, Jieun You, Ji Soo Kim, Hakchun Kim, Jungwon Park, Jihwan Song, Inhee Choi

**Affiliations:** 1 Department of Life Science, University of Seoul, 163 Seoulsiripdaero, Dongdaemun-Gu, Seoul, 02504 Republic of Korea; 2 Department of Applied Chemistry, University of Seoul, 163 Seoulsiripdaero, Dongdaemun-Gu, Seoul, 02504 Republic of Korea; 3 Department of Mechanical Engineering, Hanbat National University, 125 Dongseodaero, Yuseong-Gu, Daejeon, 34158 Republic of Korea; 4 School of Chemical and Biological Engineering, Institute of Chemical Process, Seoul National University, 1 Gwanakro, Gwanak-Gu, Seoul, 08826 Republic of Korea; 5 Center for Nanoparticle Research, Institute for Basic Science (IBS), Seoul, 08826 Republic of Korea

**Keywords:** Hybrid nanogel, Light-responsive delivery, Photothermal conversion, Plasmonic nanoparticles

## Abstract

**Background:**

Spatiotemporal regulation is one of the major considerations for developing a controlled and targeted drug delivery system to treat diseases efficiently. Light-responsive plasmonic nanostructures take advantage due to their tunable optical and photothermal properties by changing size, shape, and spatial arrangement.

**Results:**

In this study, self-integrated plasmonic hybrid nanogels (PHNs) are developed for spatiotemporally controllable drug delivery through light-driven conformational change and photothermally-boosted endosomal escape. PHNs are easily synthesized through the simultaneous integration of gold nanoparticles (GNPs), thermo-responsive poly (*N*-isopropyl acrylamide), and linker molecules during polymerization. Wave-optic simulations reveal that the size of the PHNs and the density of the integrated GNPs are crucial factors in modulating photothermal conversion. Several linkers with varying molecular weights are inserted for the optimal PHNs, and the alginate-linked PHN (A-PHN) achieves more than twofold enhanced heat conversion compared with others. Since light-mediated conformational changes occur transiently, drug delivery is achieved in a spatiotemporally controlled manner. Furthermore, light-induced heat generation from cellular internalized A-PHNs enables pinpoint cytosolic delivery through the endosomal rupture. Finally, the deeper penetration for the enhanced delivery efficiency by A-PHNs is validated using multicellular spheroid.

**Conclusion:**

This study offers a strategy for synthesizing light-responsive nanocarriers and an in-depth understanding of light-modulated site-specific drug delivery.

**Graphical Abstract:**

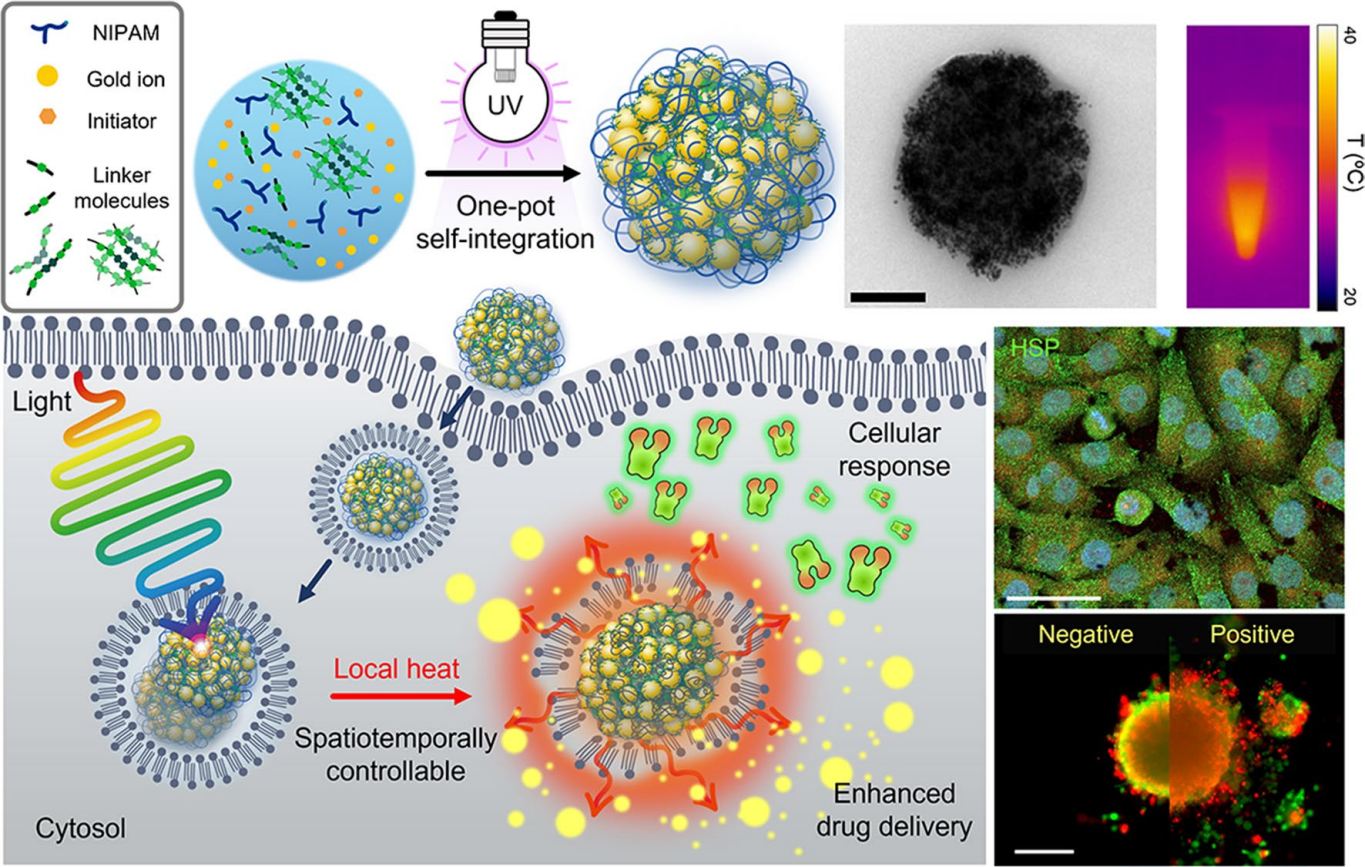

**Supplementary Information:**

The online version contains supplementary material available at 10.1186/s12951-023-01935-x.

## Introduction

Stimuli-responsive drug delivery systems using functional nanoparticles have garnered increased interest because they allow the efficient and controlled delivery of guest molecules (e.g., drugs, peptides, and nucleic acids) [1–3]. In the presence of stimuli, these functional nanocarriers exhibit regulated drug release by modulating their physicochemical properties as well as directly influencing the local environment. For example, differential cellular microenvironments can induce controlled delivery using modified functional nanoparticles resulting from the response to endogenous stimuli, including local pH [4], reactive oxygen levels [5, 6], and the presence of a specific enzyme [7]. However, these endogenous stimuli have a relatively low spatial resolution, which could lead to the failure of precise delivery owing to the difficulty in distinguishing between the targeted spot in vivo and other spots with similar conditions [8, 9]. This problem may be avoided with the use of switchable exogenous stimuli such as light [10, 11], temperature [12], electromagnetic fields [13], and ultrasound [14] with high spatial controllability after recognition or internalization in the target cells as an alternative strategy for improving delivery accuracy.

Most nanoparticles that interact with cells could be internalized through the vesicle trafficking pathway because this system effectively encapsulates exogenous materials (e.g., nanoparticles, viruses, and macromolecules) in endocytic vesicles [15, 16]. The innate cellular defense system subjects external materials such as nanoparticles to degradation in the endo-lysosome vesicles; however, intracellular delivery does not occur until after they exit the vesicle. Thus, designing nanoparticles that can escape from the endo-lysosomal vesicles after cellular uptake is essential for improving drug delivery efficiency. In addition to vesicular rupture or particle swelling in response to cytosolic environments such as pH and osmotic pressure [17–19], exogenous triggers for membrane melting or poration may also be employed to effectively open the vesicular membranes. For example, the use of pulsed lasers and/or light-responsive plasmonic metal nanoparticles can rupture endosomal vesicles rapidly by generating vapor nanobubbles or photothermal heating [20–22].

Additionally, light-mediated drug delivery systems are useful for achieving local delivery because they have a good spatiotemporal resolution allowing intuitive and switchable local stimulation and controllable parameters such as wavelength, power, illumination time, and area [23–25]. In this regard, functional nanomaterials permitting light-responsive conformational changes [26, 27], singlet oxygen generation [28, 29], and photothermal conversion [30] have become useful tools to spatiotemporally manipulate drug delivery. Among them, plasmonic gold nanoparticles (GNPs), which can be synthesized in various shapes, sizes, and spatial arrangements, are widely utilized for light-responsive drug delivery and manipulation because of their biocompatibility and optical tunability [31–33]. Since GNPs can easily control heat generation levels by modulating the aforementioned properties, they can be applied to local delivery systems with minimal side effects [34–36]. Moreover, these unique properties can be incorporated into various functional polymeric structures, such as stimuli-responsive hydrogels which exhibit reversible conformational changes to regulate the drug-release kinetics, to form hybrid structures with improved drug delivery efficiency. Poly (*N*-isopropyl acrylamide) (PNIPAM) is a representative thermosensitive polymer that has been frequently utilized because of the ability to change its morphology via sharp coil-to-globule transition and sequential phase separation above a lower critical solution temperature (LCST, approximately 32 °C) [37, 38]. These conformational changes of PNIPAM according to the temperature could be ideally applicable in entrapping drugs inside the polymeric structure and releasing drugs. However, the innate poor mechanical property of the PNIPAM polymeric network in the swollen state is limited to functioning as a robust drug carrier [39, 40]. This problem could be overcome by introducing metallic nanoparticles to reinforce the mechanical strength in the swollen state [41]. The metallic nanoparticles can be integrated into the polymeric structures through chemical modification of the polymeric building blocks [42, 43] and stepwise integration during polymerization [44, 45]. However, these approaches have several limitations including low yield, difficulty in purification, and multi-step synthesis.

**Fig. 1:**
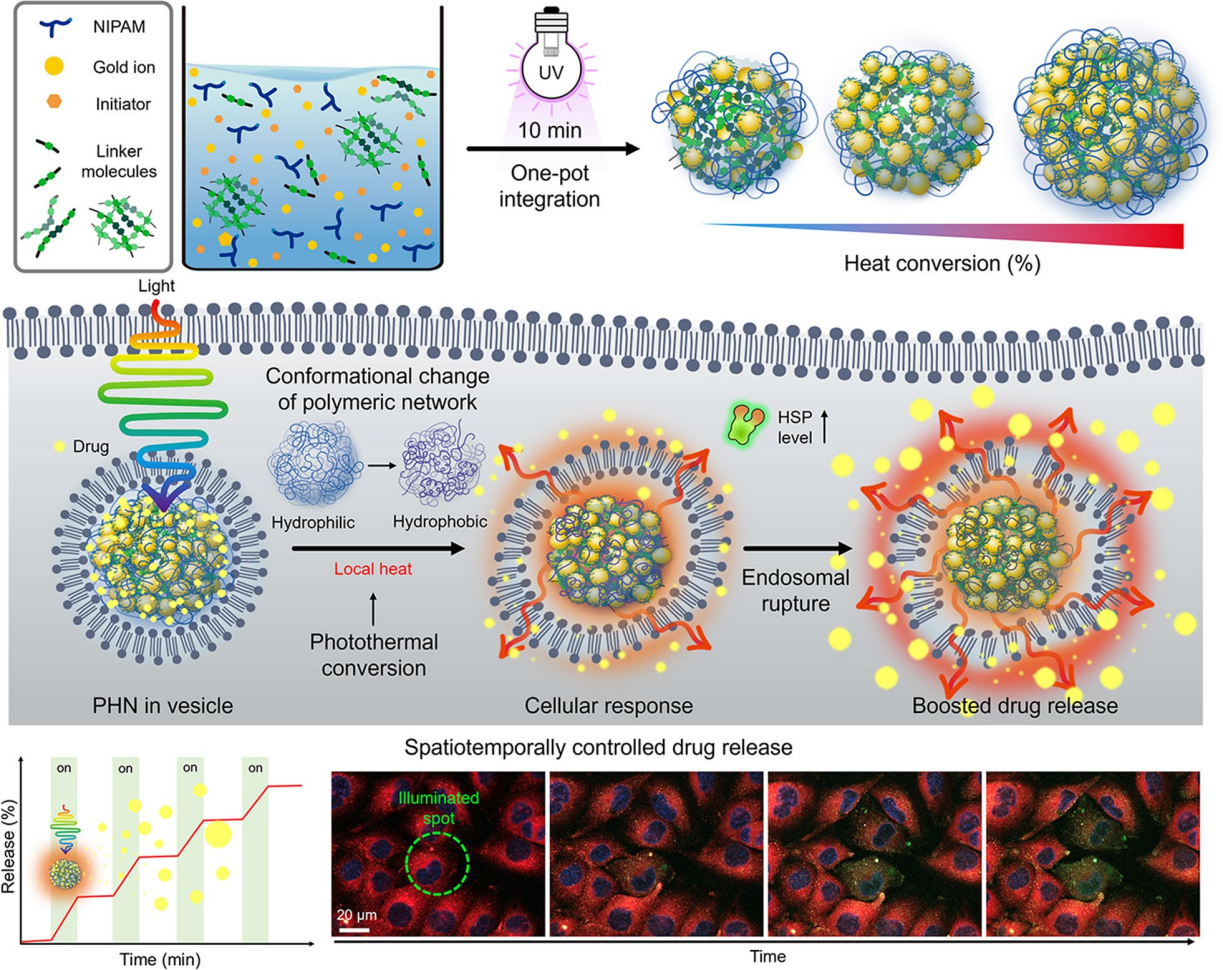
Overall schematic illustration of spatiotemporally controlled delivery through photothermally-driven drug release and facilitated the endosomal escape of light and temperature-responsive plasmonic hybrid nanogels (PHNs)

In this study, we developed light-responsive plasmonic hybrid nanogels (PHNs) for spatiotemporally controlled drug delivery via photothermally driven conformational changes and achieved enhanced drug delivery through locally boosted endosomal escape (Fig. 1). The PHNs were prepared by the simultaneous integration of light-absorbing GNPs, heat-responsive *N*-isopropyl acrylamide (NIPAM), and linker molecules during radical polymerization. By adjusting the sizes of the PHNs and altering the integration density of the GNPs by changing the linker molecules, the PHNs could be optimized to maximize their light-responsive properties. Photothermally driven conformational changes of PHNs facilitate on-demand drug delivery into cells. More specifically, this feature enables controlled drug release as well as cellular components-specific stimulation, which could permit an effective treatment using PHN itself. Thus, understanding the behavior of cellular-internalized PHNs under light illumination would elucidate the accelerated drug delivery in a spatially controlled manner. Finally, enhanced drug penetration by PHNs under light illumination can be assessed using three-dimensional cellular spheroids.

## Results and discussion

### Synthesis of plasmonic hybrid nanogels (PHNs) via photo-initiated one-pot polymerization

**Fig. 2:**
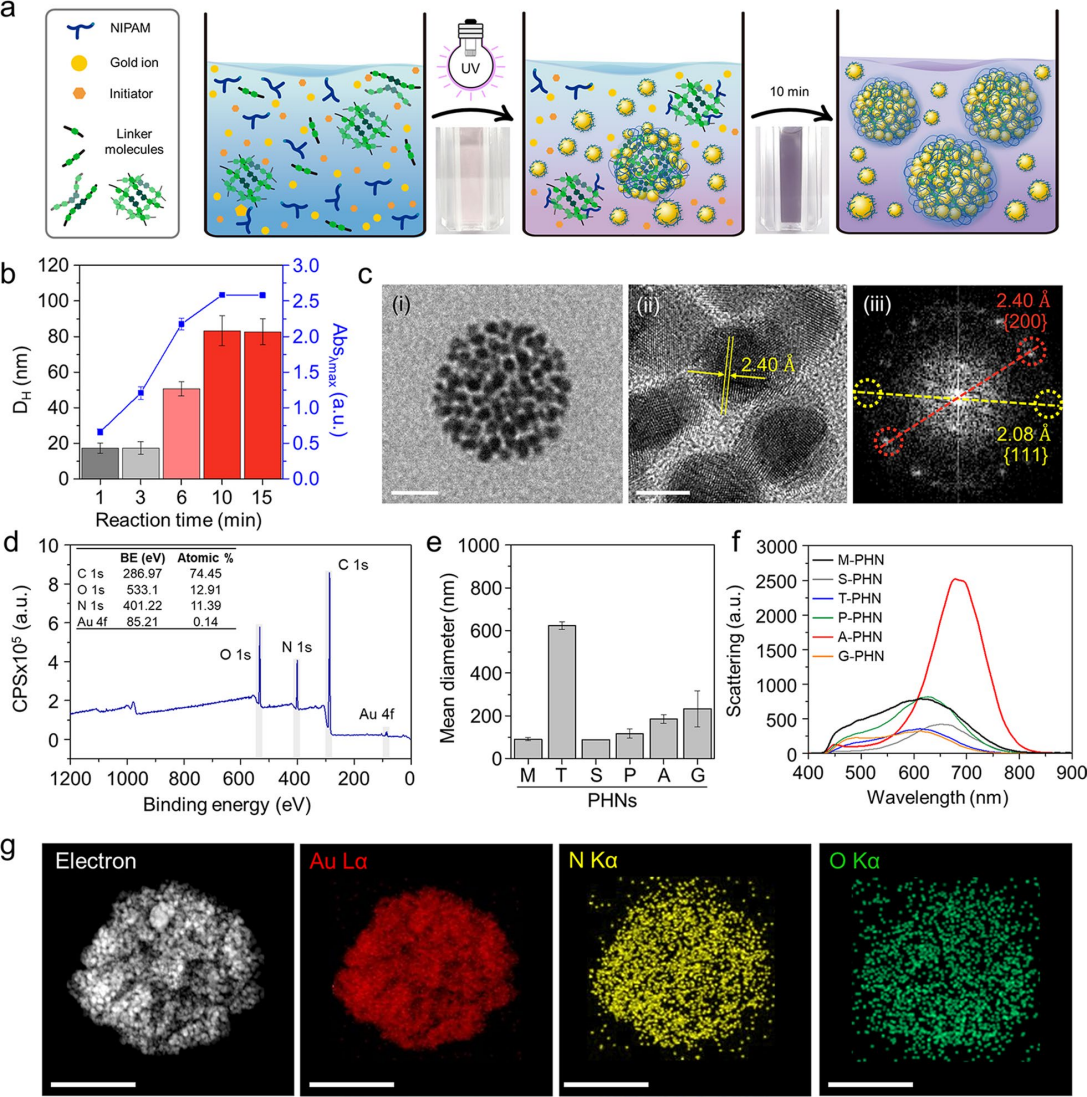
One-pot synthesis of PHNs and their physicochemical properties. **a** Schematic illustrations for simple one-pot fabrication procedure of PHNs. **b** Optimization of the reaction time by monitoring the hydrodynamic diameter and the peak absorbance (λ_max_ = 530 nm) of PHNs. **c** Electron microscopy (EM) analysis of MBA-linked PHN (M-PHN). (i) Field emission-transmission EM (TEM) image of a single M-PHM (Scale bar: 50 nm), (ii) High resolution (HR)-TEM image of the lattice structure of GNPs (Scale bar: 5 nm), and (iii) the diffraction patterns of GNPs in an M-PHN. **d** Wide scan X-ray photoelectron spectra of alginate linked-PHN (A-PHN). **e** The hydrodynamic diameters of PHNs synthesized with various linker molecules. **f** Averaged scattering spectra of various species of PHNs (n = 40). **g** Energy dispersive spectroscopy mapping results of A-PHN using HR-TEM. Elemental mapping images for Au, N, and O atoms (Scale bar: 100 nm)

To obtain PHNs composed of light-responsive gold nanoparticles (GNPs) and thermo-responsive polymeric nanogels, we employed a photo-initiated one-pot synthesis method. The reaction mixture was prepared by blending a thermo-responsive monomer (i.e., NIPAM), several linker molecules (Additional file 1: Table S1), a gold ion precursor (i.e., HAuCl_4_), and a photoinitiator (i.e., Darocur® 1173). Under exposure to 365 nm light (1.2 W/cm^2^), the reaction components quickly formed a globular structure through radical polymerization of the monomers and simultaneous self-integration of reduced gold ions into the polymeric network (Fig. 2a). The optimal condition for GNP synthesis was determined by altering the concentration and mixing ratio of the gold precursor and photoinitiator (Additional file 1: Fig. S1). After 10 min of illumination, small-sized ligand-free GNPs less than 10 nm in size were successfully formed through ion reduction by the free radicals generated from the photoinitiator [46, 47]. During the polymerization of NIPAM and linkers, the GNPs that were readily synthesized and integrated with the polymeric network via weak interactive forces between the GNP and hydrophilic moieties of the polymeric networks (i.e., –OH, –NH, etc*.*) [48, 49]. Consequently, simultaneous self-integration was achieved to form hybrid nanogels composed of a polymeric network (i.e., PNIPAM) and GNPs.

The reaction time for the synthesis of PHNs was optimized by monitoring the hydrodynamic diameter and absorbance at the peak at each time point (Fig. 2b). After sampling small aliquots, an equivalent volume of deionized (DI) water was added to the products to terminate the free radical-mediated reaction. When *N,N*′-methylene bisacrylamide (MBA) was used as a linker molecule, the size of the resulting MBA-linked PHN (M-PHN) rapidly increased and reached a plateau within 10 min. Compared to the early-stage aliquots, the matured M-PHNs exhibited a monodisperse peak (Additional file 1: Fig. S2). The diameters of the PNIPAM nanogels without GNPs increased to 400 nm as the reaction time increased (Additional file 1: Fig. S3); however, the M-PHNs reached 80 nm within the same reaction time. A difference was observed in the growth pattern between that of PNIPAM nanogel and PHNs; this could be attributed to the effect of the consumption of radicals during GNP formation on the growth of the PHNs. Moreover, the absorbance spectra of the PHNs broadened and shifted to a longer wavelength compared with that observed in the reaction with gold ions only (Additional file 1: Fig. S4). Thus, GNPs in the M-PHNs were well-incorporated into the polymeric networks during the reaction. The resulting M-PHNs exhibited relatively good stability under repetitive cycles of thermal stress than that of the PHNs without linker molecules (Additional file 1: Fig. S5). This indicates that GNPs reinforced the structural stability of the PHNs by being anchored to the polymeric networks and regulating the growth kinetics of the nanogels.

The TEM images in Fig. 2c(i) show the representative morphology of the M-PHNs exhibiting GNPs embedded into the globular hybrid nanogel structure. As shown in the high-resolution image (Fig. 2c(ii)), sub-10 nm GNPs were assembled with a small gap. The typical lattice length and diffraction patterns for the assembled GNPs revealed a strong correspondence with the metallic GNPs, which have a face-centered-cubic facet (Fig. 2c(iii)) [50]. Unlike the GNPs, the polymeric network of the PHNs was not visible in the TEM images, which might be attributed to the thinness of the layer and low electron density. X-ray photoelectron spectroscopy (XPS) measurements were performed on the outermost layer of the PHNs to characterize the polymeric layer of the PHNs with precision. The wide-scan survey spectrum of the PHNs showed peaks corresponding to C_1s_, N_1s_, O_1s_, and Au_4f_ at the characteristic binding energies (Fig. 2d). The relative atomic percentage of Au was almost indiscernible because the surface of the GNPs was mostly covered with thin polymeric layers. Nonetheless, the metallic Au^0^ peaks at 88.28 eV and 84.58 eV were measured from the narrow scan (Additional file 1: Fig. S6). This indicates that the Au ions were successfully reduced to metallic Au during polymerization. Moreover, the peak shifted slightly to higher binding energy, indicating that the GNPs were embedded in the polymeric network through weak interactive forces (e.g., Van der Waals, dipole–dipole, induced dipole, etc*.*) [46, 47, 49, 51, 52]. In the C_1s_ region, the spectrum was deconvoluted into three peaks (i.e., 284.78 eV for the C–C peak, 286.2 eV for the C–N peak, and 287.48 eV for the C=O), which indicate the presence of PNIPAM structures on the Au surface. In addition, the existence of PNIPAM was double-checked by observing at 532 eV for the C=O peak in the O_1s_ spectrum. These XPS results indicate that the GNPs were successfully entrapped inside the thin PNIPAM-based nanogels.

Since the light-responsive property is closely related to the absorption and scattering cross sections [53], the integration density of GNPs in the PHN structure or the diameters of PHN should be further optimized. For this purpose, five kinds of other linker molecules, tryptophan (T), sucrose (S), polyethylene glycol diacrylate (P), alginate (A), and gelatin(G) were tested to control the density of the GNPs and the size of the PHNs. Figure 2e shows the difference in the diameter of the synthesized PHNs, which might be attributed to the different physicochemical properties of the linker molecules such as hydrodynamic diameters, functional groups, and the presence of hydrophilic pockets. The sugar-ring structure is known to stabilize the GNPs owing to their high hydrophilicity; hence, the structural stability of PHNs can be improved by embedding GNPs into additional polysaccharides [47, 51, 52]. As expected, both the density of the GNPs and the diameter of the PHNs depended on the linker molecules (Additional file 1: Fig. S7). In the case of M-PHN, P-PHN, and A-PHN, smaller GNPs (i.e., less than 10 nm) tended to be more closely packed in the structures. In particular, the PHN fabricated with alginate as the linker (i.e., A-PHN) exhibited the highest density of the small GNPs. Consequently, the absorbance peaks shifted to longer wavelengths upon changing the linker molecules (Additional file 1: Fig. S8a). Although the PHNs with large-sized GNPs (e.g., T-PHN and S-PHN) had red-shifted absorption bands, they showed poor colloidal stability (Additional file 1: Fig. S8b), whereas the A-PHN, P-PHN, and G-PHN were stable after overnight storage at room temperature (i.e., 25 °C). Moreover, A-PHN exhibited the highest scattering peak at approximately 700 nm (Fig. 2f), which could be attributed to the large optical cross-sectional area induced by the high density of GNPs. The molecular weight (MW) of A-PHN was estimated by measuring static light scattering. As shown in Additional file 1: Fig. S9, the measured MW of A-PHN was found to be 1.04 × 10^7^ Da, whereas those of PNIPAM and PNIPAM-alginate exhibited 2.53 × 10^5^ and 4.24 × 10^6^ Da, respectively. The relatively high MW of the A-PHN is attributable to the GNP integration into the polymeric nanogels. Moreover, lyophilized A-PHN exhibited superior water solubility of up to 30 mg/mL (Additional file 1: Fig. S10). Based on these results, A-PHN was selected for further characterization and drug delivery applications.

To verify the existence of the alginate, we used calcium ions, which are well-known gelation linkers of alginate-based hydrogels and form a calcium-alginate egg-box structure [54]. As shown in Additional file 1: Fig. S11, alginate-containing dispersions (i.e., A-PHN and alginate only) formed hydrogels in the presence of Ca^2+^, whereas gel formation was not observed for M-PHN. The Ca^2+^-induced formation of the purple-colored gel observed in the case of the A-PHN indicated that alginate molecules were successfully incorporated into the GNPs and PNIPAM network because the alginate-only hydrogel exhibited a white color. Elemental mapping (Fig. 2g) and energy-dispersive X-ray spectroscopy results (Additional file 1: Fig. S12) for A-PHN also showed the successful integration of each component. These results confirmed that high-density GNPs are embedded into alginate-linked PNIPAM nanogels, and the resulting A-PHN possesses a large optical cross-sectional area to the incident light.

### Local heat generation by photothermal conversion of GNPs in the PHNs

**Fig. 3:**
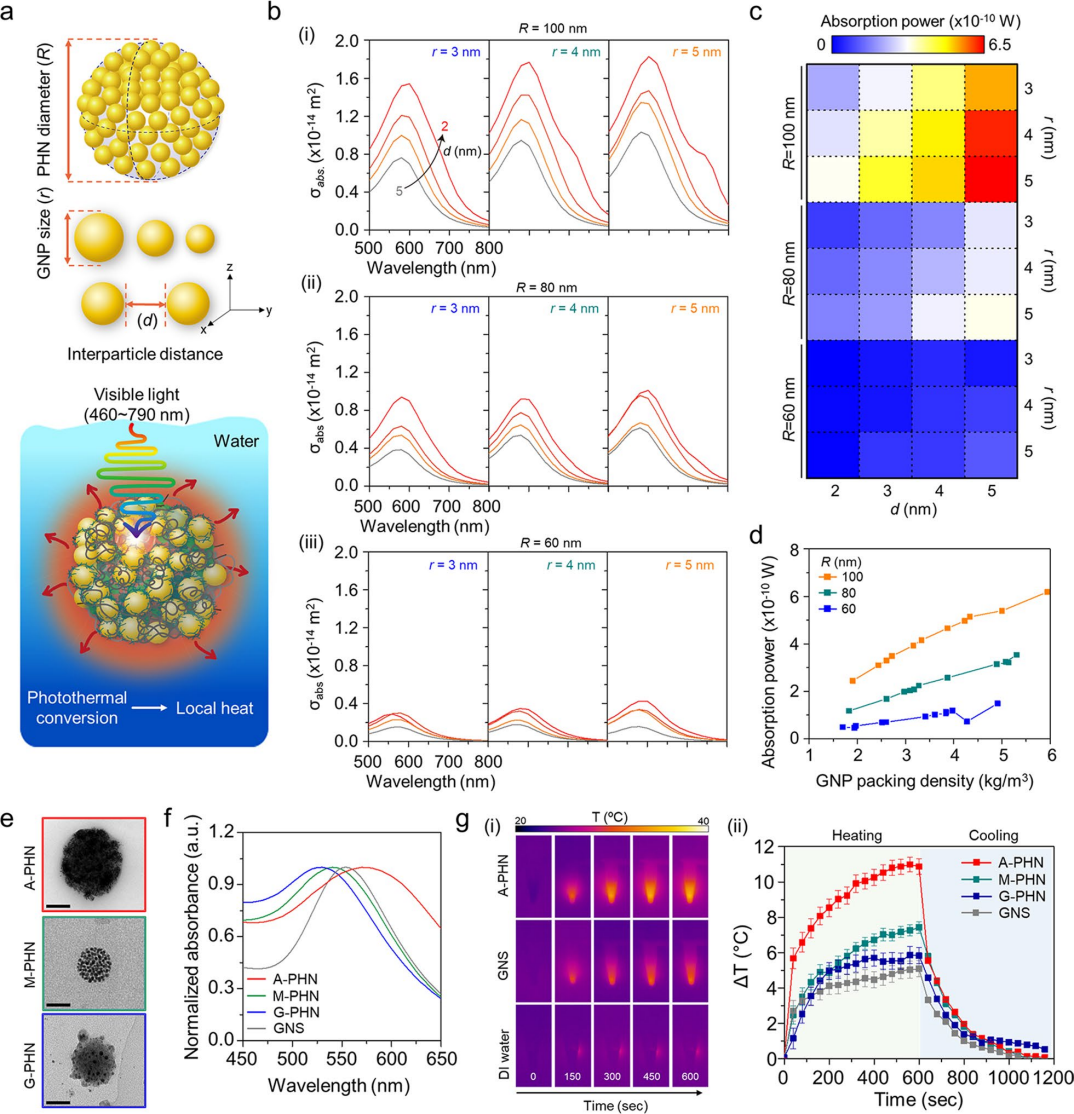
Evaluation of local heat generation from the photothermal conversion of GNPs in PHNs under light illumination. **a** Schematics of computational simulation parameters (i.e., *R*, *r*, and *d*) and modeling environment. **b** Computational simulation for the absorbed cross-section of PHNs according to the diameters of single GNP (*r*), from left (*r* = 3) to the right (*r* = 5) for (i) *R* = 100, (ii) *R* = 80, and (iii) *R* = 60. **c** Absorption power map profile for the degree of local heat generation for various values of *R*, *r*, and *d*. **d** GNP packing density-dependent absorption power plots at 550 nm by changing the *R*. **e** Representative TEM images of the PHNs with various integration densities of GNPs (Scale bar: 50 nm). **f** Normalized absorbance spectra of the PHNs and GNS. **g** Monitoring of heat generation by 100 µL PHNs and 80 nm GNS. (i) Infrared thermal images. (ii) Thermal elevation curves with heating and cooling period (n = 3)

A wave optics simulation was performed to predict the local heat generation from the PHNs under light illumination by calculating the absorption power of GNPs in the PHNs. First, it was considered that a single PHN structure was immersed in water, and the light at an intensity of 3.5 W/cm^2^ was continuously irradiated at wavelengths from 460 to 790 nm. For this modeling, the GNPs were arranged at regular intervals based on the GNP size (*r*) and interparticle distance between the GNPs (*d*) at the differential PHN diameter (*R*) (Fig. 3a and Additional file 1: Fig. S13). The optical properties of the PHN structure were evaluated at different values of *r* (3, 4, and 5 nm), *d* (2, 3, 4, and 5 nm), and *R* (60, 80, and 100 nm). The number of GNPs was set to the maximum number that could be enclosed in the PHN. Figure 3b shows the absorption cross-section (*σ*_abs_) of the PHN according to the wavelength of the incident light. The spectrum of *σ*_abs_ varies according to *r* and *d*. When the radius of the PHN was 100 nm, *σ*_abs_ increased as *r* increased, and *d* decreased. These results show a similar tendency as *R* changed from 80 to 60 nm. The absorption power was calculated at a wavelength of 550 nm, where PHN showed the maximum *σ*_abs_. As shown in Fig. 3c, the absorption power also increased as *r* increased, and *d* decreased when *R* = 100 nm. Moreover, the absorption power at 550 nm increased with the GNP packing density in the PHNs (Fig. 3d). Therefore, a small number of GNPs with larger *r* values show higher absorption power than a large number of GNPs with smaller *r* when the interparticle distance was the same. As a result, the increased density of the proximate GNPs in the large PHNs induced more intensive heat generation.

To experimentally validate this prediction, three types of PHNs (i.e., M-PHN, A-PHN, and G-PHN), which have different GNP densities, were prepared with the same concentration (i.e., 1 mg/mL, optical density = 1OD at peak) as shown in Fig. 3e. Considering the similar size and absorption peak wavelength of the PHNs, an 80-nm gold nanosphere (GNS) was used as a control to evaluate the photothermal conversion efficiency of the PHNs (Fig. 3f). As shown in Fig. 3g, the solution temperature of the colloidal A-PHN drastically increased by 11 °C on exposure to a 532 nm laser (3.5 W/cm^2^). Notably, the temperature increment depended on the density of the GNPs (i.e., A-PHN > M-PHN > G-PHN). In particular, the heat increment level was twofold higher for the A-PHN, which had high-density GNPs, compared with that of the GNS. Furthermore, the photothermal conversion efficiency (*η*) of the GNS and PHNs was calculated to quantitatively compare the heat generated by the colloids [30, 35]. The rate of heat transfer was measured by removing the light source and monitoring the decrease in temperature (see Additional file 1 for detailed calculations). From the calculations, *η* was found to be 15.69, 8.54, and 3.99% for A-PHN, M-PHN, and GNS, respectively (Additional file 1: Fig. S14). A higher *η* for A-PHN than for M-PHN indicates that the packing density of GNPs in the PHN is crucial for effectively generating heat in response to light. For lyophilized PHN powder, temperature increments prominently occurred up to 80 °C, even under exposure to laser at a relatively low power density (532 nm, 0.8 W/cm^2^). This can be attributed to a high-density state in the powder than in the colloidal state of the PHNs (Additional file 1: Fig. S15). Temperature elevation higher than 20 °C was achieved in the A-PHN colloids by increasing the illumination time to 1800 s (Additional file 1: Fig. S16) using a commercial LED (0.8 W/cm^2^), which emitted light with a broad wavelength (480–700 nm).

### Photothermally-driven conformational changes of PHNs

**Fig. 4:**
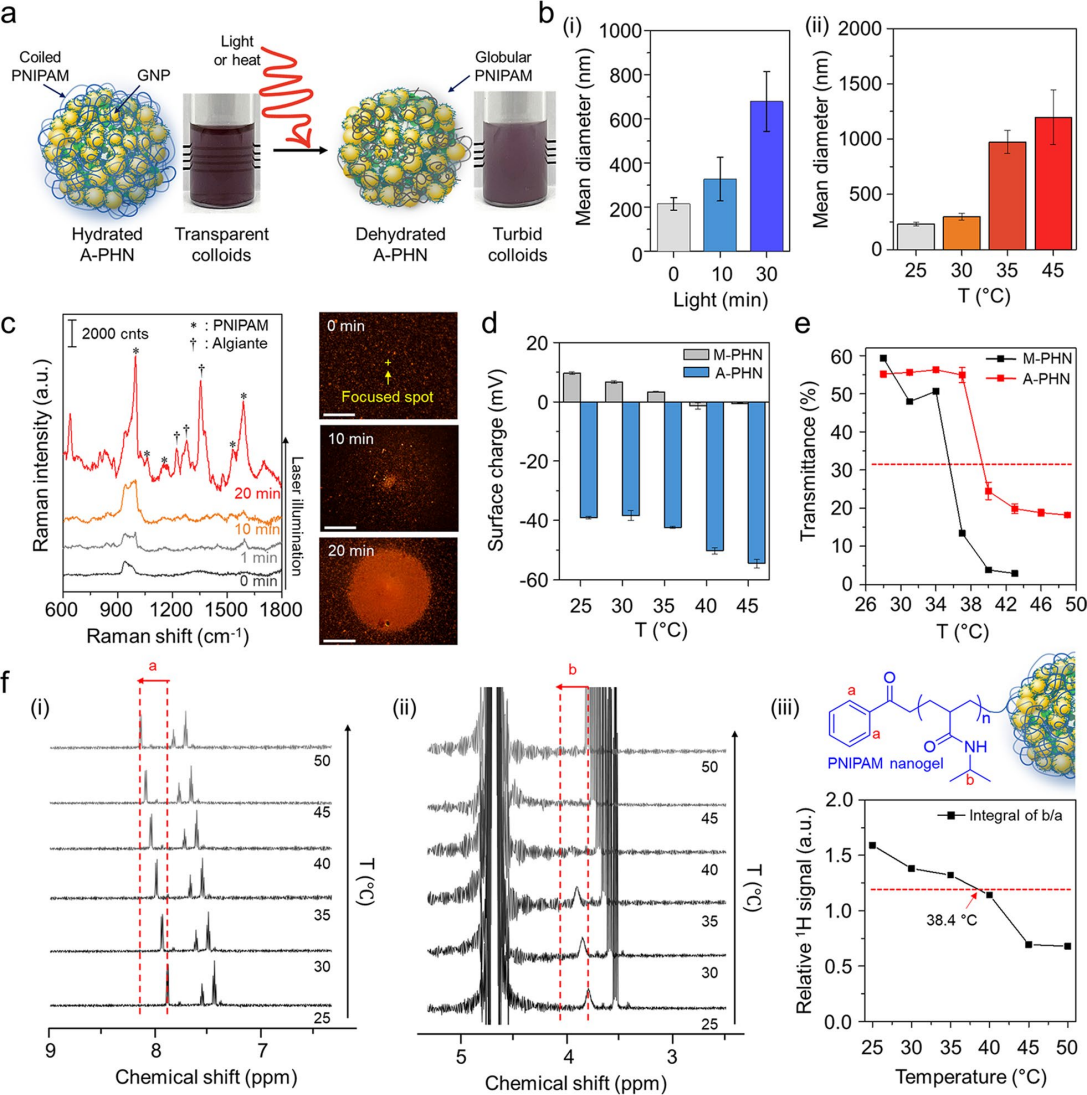
Investigation of photothermally-driven conformational changes in PHNs. **a** Schematics for dehydration procedure of A-PHN under the stimuli (i.e., light or heat). **b** Hydrodynamic diameters of A-PHN dispersion according to changing (i) light exposure time and (ii) solution temperature. **c** In-situ Raman measurements of A-PHN during the continuous laser illumination, and dark-field images of assembled A-PHN at one spot (Scale bar: 20 µm). **d** Surface charges of A-PHN and M-PHN by increasing the solution temperature. **e** Monitoring of transmittance using A-PHN and M-PHN by increasing the solution temperature. **f** Temperature-dependent ^1^H-NMR of A-PHN dispersed in D_2_O. (i, ii) Cropped spectra corresponding to PNIPAM nanogel-specific bonds denoted as *a* and *b*. (iii) The temperature-dependent relative integrals of the typical proton signals from spectra

As the resonant laser was capable of increasing the solution temperature by more than 10 °C within 10 min, we supposed that it was sufficient to induce the conformational changes in the PNIPAM network at A-PHN. The heat- or light-responsive properties of hydrated A-PHNs in an aqueous solution were evaluated by increasing the solution temperature and light exposure time. Under suitable stimulation, the A-PHN structure became unstable owing to the increased hydrophobicity of the PNIPAM side chains, which led to interparticle aggregation (Fig. 4a). The hydrodynamic diameters of A-PHNs gradually increased upon exposure to both light (Fig. 4b(i)) and heat (Fig. 4b(ii)). This indicates that either local or global heat can induce conformational changes in the A-PHNs. Since the hydrophilic PNIPAM chains dehydrate as the solution temperature exceeds the LCST [37, 38], their hydrophobic isopropyl branch and backbone form a globular structure owing to the hydrophobic interaction, which results in the agglomeration of A-PHNs in colloids.

To confirm the conformational changes of the PNIPAM units in A-PHN, Raman measurements were performed to distinguish between the hydrated and dehydrated status. As the alginates were linked with PNIPAM in the A-PHN, the shrinkage of the PNIPAM chain could be preserved with the introduction of Ca^2+^ in the turbid state (i.e., dehydrated A-PHN gel). As shown in Additional file 1: Fig. S17, the dehydrated A-PHN gel presented fingerprint peaks for alginate (*ν* = 1240, 1350, and 1440 cm^−1^) and hydrophobic moieties of PNIPAM (i.e., C–C ring stretching and breathing, *ν* = 980, 1050, and 1580 cm^−1^), unlike the hydrated gel. This can be interpreted to indicate that the alginate-linked PNIPAM network in the PHN was proximate to the GNPs by conformational changes induced due to dehydration. In Fig. 4c, the dark-field scattering images show the spatially-controlled formation of agglomerates under light illumination. With increasing light exposure time, the size of the agglomerate increased, and the resulting Raman signal for the A-PHN dynamically changed to a spectral pattern similar to that of the dehydrated gel. This means that the photothermal conversion of GNPs can induce conformational changes in the thermo-responsive PNIPAM at A-PHN. Furthermore, the change in the surface charge with increasing temperature clearly shows evidence of such changes in the PNIPAM structure at the surface of A-PHN (Fig. 4d). The surface charge of A-PHN was initially negative (c.a. − 39 mV) owing to the negatively charged alginate, and it changed to a more negative value of − 54 mV as the solution temperature increased to 45 °C. In the case of M-PHN, the same tendency toward negative charges was observed. Subsequent investigations focused on finding an optimal temperature range for the conformational changes in PHNs. Based on the change in transmittance and turbidity of the colloidal PHNs with increasing temperature, LCST values of approximately 38.4 °C and 35.6 °C for the A-PHN and M-PHN, respectively, were obtained (Fig. 4e). These temperatures were higher than that obtained for PNIPAM-based polymers in general (i.e., 32–33 °C). This is probably due to additional intermolecular interactions (i.e., hydrogen bonds) between PNIPAM and alginate or GNPs in A-PHN. Moreover, the ^1^H-NMR study using A-PHN, which is dissolved to 10 mg/mL in D_2_O, showed a temperature-dependent peak shift to downfield, which is strong evidence of the conformational changes in the PNIPAM nanogel (Fig. 4f and Additional file 1: Fig. S18) [55]. Since those changes of A-PHN occur close to 38.4 °C, which is an easily achievable range through the photothermal conversion of GNPs, photothermally triggered spatiotemporal controllability of A-PHN in the cellular delivery can be easily accomplished.

### Photothermally-driven spatiotemporally controlled drug delivery using A-PHN

**Fig. 5:**
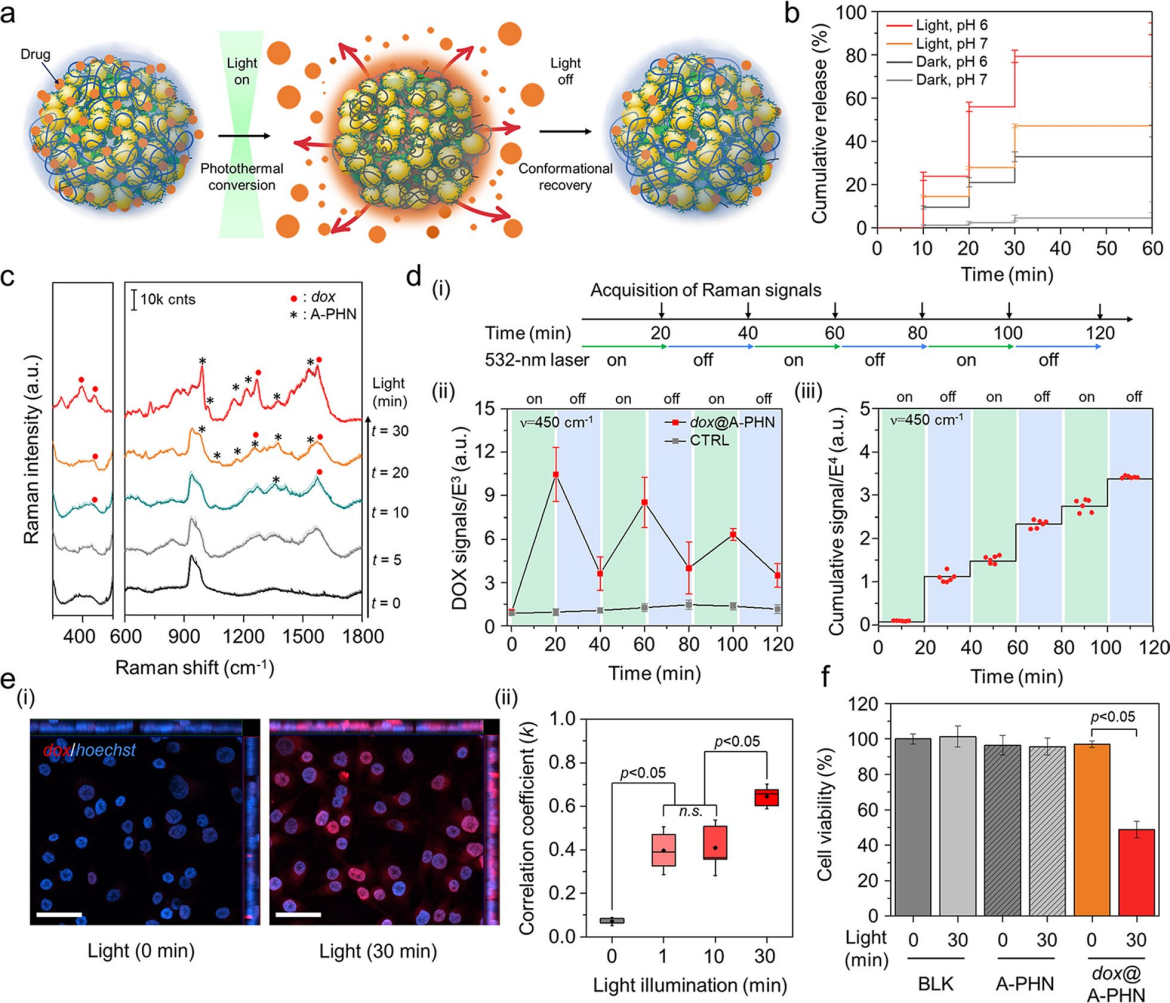
Photothermally-driven spatiotemporally controlled drug delivery using A-PHN. **a** Schematic mechanisms of drug release from a dehydrated A-PHN by light illumination. **b** Cumulative *dox* release under light exposure by measuring the absorbance of free *dox* using different pH conditions. **c** Time-coursed Raman spectra with continuous light illumination. **d** Monitoring the Raman spectra of released *dox* under temporally controlled light modulation. (i) Schematics for the discrete light illuminating experiments. (ii) Switchable release profile of *dox*@A-PHN under laser on/off conditions. (iii) Cumulative *dox* signal kinetics under discrete light illumination (n = 6). **e** Observation of light-triggered *dox* delivery using A-PHN. (i) Fluorescent colocalization images of released *dox* within the nucleus. The red color indicates the *dox*, and the blue indicates the nucleus. The scale bar is 50 µm. (ii) Colocalization degree monitoring by Pearson’s coefficient with light exposure time (n = 3). **f** Cell viability test using MTT assay of A-PHN and *dox*@A-PHN with/without light exposure (n = 5)

To verify the controlled drug release by photothermally driven conformational changes of the A-PHN (Fig. 5a), two model drugs, doxorubicin (*dox*) and paclitaxel (*ptx*), were loaded into the A-PHN during the calcium-induced gelation method. Briefly, 1 mg/mL A-PHN was mixed with each drug solution (i.e., 200 µM *dox* and 1 µM *ptx*) and subsequently added 10 mM CaCl_2_. Then, they incubated for 2 h to check the drug-loaded gel formation. After being isolated from free drugs, the precipitates were re-suspended to water. The loading efficiency of *dox* was calculated to be 60% (v/v) according to the standard curve for *dox* absorbance (Additional file 1: Fig. S19). As shown in Fig. 5b, the loaded *dox* was released faster from the A-PHN in the presence of light (LED, 0.8 W/cm^2^). And, the release kinetics were dependent upon the light power density (Additional file 1: Fig. S20). Moreover, the release profile drastically increased under acidic conditions, which resulted from the loosened polymeric network at low pH (Additional file 1: Fig. S21) and the synergistic effect of protonated *dox* to boost the release [56]. Additionally, photothermally induced drug release was monitored using the intrinsic Raman signals of *dox* (Fig. 5c). According to the illumination time, the Raman signals of *dox* emerged gradually, whereas the same peaks were also observed in the drug-loaded A-PHN itself (see Fig. 4c). This attribute of the structural changes in A-PHN also occurred following the loading of *dox* with calcium ions. Moreover, a series of Raman spectra were obtained to check the temporal controllability of drug release from A-PHNs under discrete light illumination (532 nm laser, 3.5 W/cm^2^). Figure 5d(i) shows the Raman signals obtained alternatively in the presence and absence of laser illumination. The released *dox* signals drastically increased initially during the “laser on” condition (Fig. 5c and Additional file 1: Fig. S22), whereas only minimum signals were observed during the “laser off” condition. The increase in the Raman signal slowed as the amount of remaining *dox* inside the A-PHN decreased after the first sequence (Fig. 5d(ii) and (iii)). This clearly demonstrates the temporal controllability of drug release in response to light. The colloidal stability of the A-PHNs was evaluated by measuring mean diameters in various biological media including PBS, DMEM, and RPMI. As shown in Additional file 1: Fig. S23, the change in diameters was negligible for 7 days, indicating the high stability of the A-PHN in biological media. Moreover, the MTT assay was conducted to confirm the cytotoxicity of A-PHN itself and the cellular effect from photothermal conversion. As shown in Additional file 1: Fig. S24, no significant cytotoxicity of nanogels and A-PHNs even at high concentrations was observed before light exposure.

*Dox* is well-known for specifically targeting the cell nucleus; therefore, the released drugs from the A-PHN would travel rapidly into the nuclei. To visualize this, *dox*@A-PHN (i.e., 80 µM *dox*) was used to treat A375P melanoma cells. The cells were placed under an LED (0.8 W/cm^2^) for predetermined periods (i.e., 0, 1, 10, and 30 min). Non-light illuminating groups were placed in the dark to avoid undesired leakage of *dox* from the A-PHN. As a result, colocalization of *dox* within the nuclei was more noticeable in the cells in the 30-min light exposure group than in the cells of the control group that were incubated in the dark (Fig. 5e(i)). To quantify the delivery efficiency, Pearson's correlation coefficient (*k*) was used to examine the degree of colocalization between *dox* and Hoechst. As expected, this value tended to increase in a time-dependent manner from 0.07 (at 0 min) to 0.645 (30 min). The value of the control group was significantly lower than that of the light-exposure groups (Fig. 5e(ii) and Additional file 1: Fig. S25). A cytotoxicity test was performed under the same conditions to further confirm drug delivery efficacy. To avoid side effects on the cells from the remaining *dox*@A-PHN, the incubated cells were carefully washed and replaced with fresh media. Since *dox* does not kill the cells immediately, additional incubation was provided overnight before assaying. For the cells treated with *dox*@A-PHN under 30 min of light illumination, a significant decrease (48.9 ± 4.6%) in cell viability was observed (Fig. 5f). Notably, no significant cell death was observed in the control groups, including light exposure only without *dox*@A-PHN (101.3 ± 5.9%), *dox*@A-PHN in the dark (97.0 ± 1.8%), and A-PHN only (95.7 ± 4.8%) groups with and without light exposure.

### Photothermally facilitated the endosomal escape of cellular internalized A-PHN

**Fig. 6:**
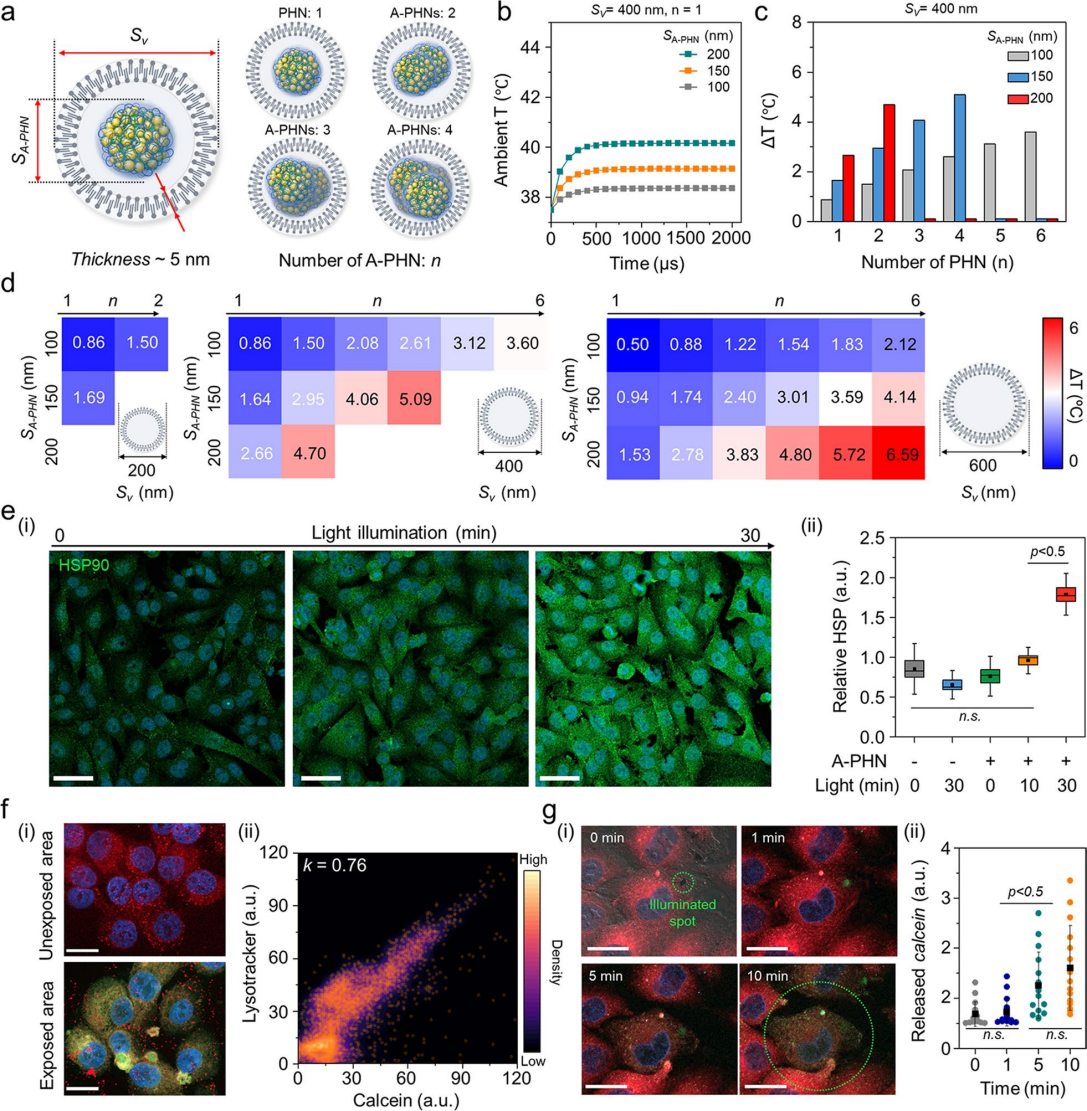
Pinpoint cytosolic manipulation and spatially controlled drug release due to light-triggered vesicle rupture in a single cell. **a** Schematics of the simulation domain consist of the endocytic vesicle that encloses the A-PHNs. **b** Computational simulation for heat elevation profile using *S*_*v*_ = 400 nm and *n* = 4 by changing the size of single *S*_*A-PHN*_ in the vesicle. **c** Mean temperature changes of the vesicle with *S*_*v*_ = 400 nm. **d** Temperature shift expectation plot by changing the number (*n*), and size (*S*_*A-PHN*_) of PHNs in the various-sized vesicle (*S*_*v*_). **e** Monitoring the cellular response (i.e., HSP expression) according to light illumination. (i) Fluorescence images for the elevated HSP under the mild LED (0.8 W/cm^2^). The scale bar is 50 µm. (ii) Comparison of relative HSP intensity (n = 5) based on illuminating time. **f** Spatially controlled delivery using *calcein*-encapsulated A-PHN by photothermally-driven vesicular rupture. (i) Fluorescence images of endo-lysosome and released *calcein* from the A-PHN at the unexposed and exposed regions (Scale bar: 20 µm). (ii) Colocalization density plot between lysotracker and *calcein* (orange dots indicate the colocalization spots). **g** Real-time monitoring of *calcein* leakage at the single cell. (i) Time-coursed confocal images by illuminating the laser (550 nm with 3.5 W/cm^2^). Red color indicates the endo-lysosomes, and green indicates the released *calcein*. The scale bar is 10 µm. (ii) Released *calcein* intensity profiles in the single cell according to the light exposure time (n.s. indicate the non-significance)

For a more nuanced understanding of the sophisticated drug delivery mechanism, we investigated the temperature distribution inside vesicles containing A-PHNs. By observing the fluorescent and dark-field scattering images, we confirmed that A-PHN with 150–200 nm in diameter could be internalized into cells via the endocytosis pathway [15, 16]. As shown in Additional file 1: Fig. S26, the diameters of vesicles including A-PHNs ranged between 200 and 800 nm and are visualized as red fluorescent dots (i.e., endo-lysosomes) and orange scattering dots (i.e., A-PHN-containing vesicles). The broad distribution of vesicle sizes can be attributed to the additional packing process during the proximate vesicle fusion in the cytosol after internalization. Based on the experimental observation of endocytosis, a simulation was performed to predict the temperature profiles by changing the number of A-PHNs in the endocytic vesicle under the light. As shown in Fig. 6a, the simulation domain consisted of a vesicle that enclosed the A-PHNs. The outer layer of the vesicle was a lipid bilayer membrane with a thickness of 5 nm, and the inside of the vesicle was assumed to be filled with water. We conducted a simulation by varying the size of the vesicles (*S*_*v*_) and A-PHNs (*S*_*A-PHN*_) and the number of encapsulated A-PHNs (*n*). In the simulation, various *S*_*v*_ (200 nm, 400 nm, and 600 nm), *S*_*A-PHN*_ (100 nm, 150 nm, and 200 nm), and *n* (1 to 6) were applied. The temperature changes inside the vesicles were calculated using the maximum absorption power obtained from the wave optics simulation as the heat source for the heat-transfer simulation. The initial ambient temperature was set to 37.5 °C, which is the common incubation temperature for cellular experiments. The average temperature inside the vesicles rapidly increased in the presence of A-PHN (< 500 µs) and could be modulated according to the changes in parameters (Fig. 6b and Additional file 1: Fig. S27). For example, the mean temperature increased from the initial temperature of 37.5 °C to 42.6 °C when *n* = 4, *S*_*v*_ = 400 nm, and *S*_*A-PHN*_ = 150 nm (Fig. 6c). The result of the heat transfer simulation (Fig. 6d) showed that A-PHNs in the endocytic vesicle could generate sufficient temperature for vesicle rupture (i.e., ΔT > 4 °C) [57–59]. These results indicate that spatially controlled drug delivery is possible after the progression of endocytic vesicle growth.

To confirm the simulation results experimentally, the cytosolic response was first monitored by applying laser illumination (532 nm, 3.5 W/cm^2^) to a vesicular A-PHN (Additional file 1: Fig. S28). After the laser treatment, bubbles were generated immediately (< 1 s) around the A-PHN, which irreversibly destroyed the cytoskeleton. This also indicates that a high level of heat was generated around the vesicles [60, 61]. Since the cells maintain homeostasis under stress conditions, the photothermal-driven cytosolic temperature changes would also influence cellular components. For example, owing to the local heat generation in cells, heat shock proteins (HSPs) can be upregulated by light exposure in the presence of A-PHN. After incubating the A-PHNs in A375P cells for 4 h, low power LED (0.8 W/cm^2^) was used to illuminate the cell. The level of HSPs markedly increased in the A375P cells with internalized A-PHN according to the illumination time (Fig. 6e(i)). As shown in Fig. 6e(ii), a drastic increase in the HSP intensity was observed in the A-PHN-internalized A375P cells with statistical significance within 30-min of light exposure. This is consistent with the observation presented in Fig. 5e, which indicates that the internalized A-PHNs could manipulate cellular responses by generating heat from the vesicles.

To effectively deliver drugs to targeted cellular sites, internalized drug-loaded carriers must escape from endocytic vesicles by staining the endosomes with wheat germ agglutinin, which binds to glycoproteins of the cell membrane, cellular internalization was validated for A-PHN through the receptor-mediated endocytic trafficking pathway [62, 63]. As shown in Additional file 1: Fig. S29, a decrease in green fluorescence indicating endosomes was prominently observed in the late endosome regions. This is well supported by the simulation result (see Fig. 6d) showing that the endosomal rupture preferentially occurs in vesicles containing multiple A-PHNs after the progression of endocytic vesicle growth. To confirm the drug release from vesicles, a membrane-impermeable fluorescent dye (i.e., *calcein,* 5 mM in DMSO) was encapsulated into the A-PHN and delivered to the cells [20, 21]. As shown in Fig. 6f(i), the green fluorescent signals corresponding to the released *calcein* were spread into the whole cytosol in the light-exposed region of the cell (550 nm laser, 3.5 W/cm^2^), whereas only red dots (i.e., lysotracker, DND-99) were observed in the unexposed area. The *calcein* signals released into the cytosol highly overlapped with the lysotracker signals after full spread (*k* = 0.76, Fig. 6f(ii)), which indicates spatially controlled drug delivery only in the light-exposed region. Moreover, we observed widespread cytosolic distribution of *calcein* within 10 min of light treatment in a single cell (Fig. 6g). This reveals that pinpoint cytosolic manipulation can be achieved quickly and A-PHN can selectively influence the vesicular membrane by local heat generation for spatiotemporally controlled drug delivery.

### Validation of enhanced drug delivery efficiency of PHNs using cellular 3D spheroids

**Fig. 7:**
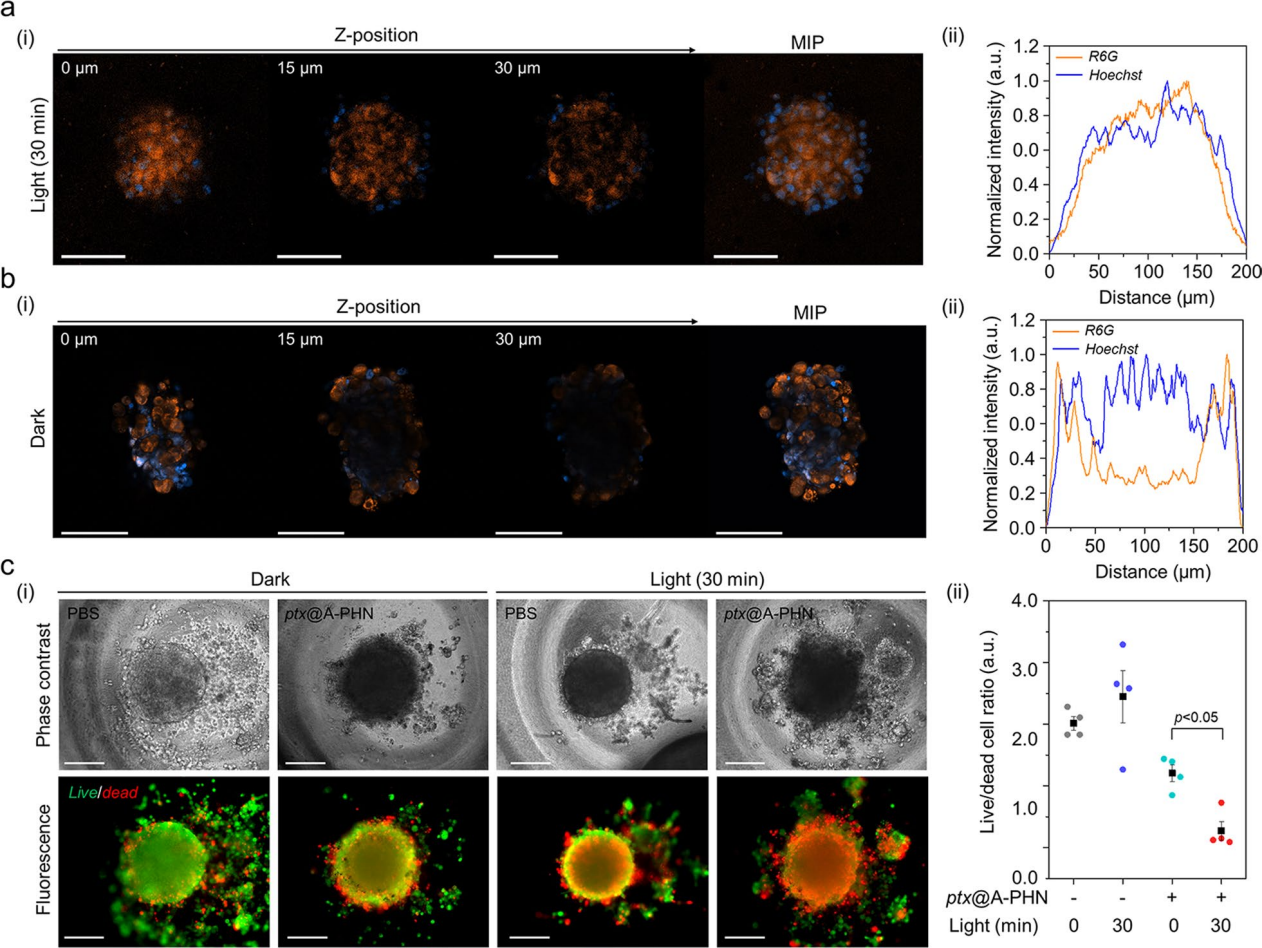
Enhanced drug delivery efficiency of the PHNs, validated using 3D cellular spheroids. Monitoring the drug penetration depth into the spheroids using R6G@A-PHN under **a** light exposure and **b** dark conditions. (i) Z-stack confocal images and maximum intensity projection (MIP) image of a single spheroid. Scale bars indicate 100 µm. The orange color indicates the R6G, and the blue color represents the nucleus in the spheroid. (ii) XY-plane profiles of fluorescent distribution in the spheroid. **c** Evaluation of delivery efficacy toward 3D spheroid using the released drug (i.e., *ptx*). (i) Live/dead cell images (Scale bar: 200 µm) and (ii) comparisons of the live/dead cell ratio from individual spheroids (n = 5)

To further examine the enhanced drug delivery efficiency using A-PHNs, a three-dimensional (3D) spheroid of cells, which mimics the microenvironments of the physiological system, was utilized. The delivery efficiency was evaluated based on the drug penetration depth and the ratio of live/dead cells. The penetration depth profiles were evaluated using a fluorescent dye (i.e., rhodamine 6G, R6G) loaded into the A-PHN (i.e., R6G@A-PHN, 100 nM R6G). The results showed that R6G was successfully diffused into the deeper regions of the spheroid in the light-exposed group (Fig. 7a, 532 nm laser, 3.5 W/cm^2^), whereas the spheroid in the dark exhibited a limited penetration depth (only observable near the outer surface, Fig. 7b). This is attributable to the generation of a temperature gradient from the A-PHN under light illumination, which would induce the rapid diffusion of drug molecules released from the A-PHN. Moreover, since the drugs released from the A-PHN can easily involve cell–cell communications to adjacent cells in the spheroid [64, 65], deeper penetration of the drugs would be achievable.

In the live/dead cell assay, the non-fluorescent anticancer drug paclitaxel (i.e., *ptx*) was used to avoid the overlapping of fluorescence during the assay. Prior to conducting spheroid experiments, phenotypic changes in the two-dimensional (2D) cell culture model were assessed by checking the *ptx*@A-PHN to determine whether the released drugs worked accurately (Additional file 1: Fig. S30). *Ptx*-induced apoptotic nuclear fragmentation in A375P cells was observed in both *ptx*-and *ptx*@A-PHN (with light exposure)-treated groups, whereas no change was observed in the A-PHN treatment group. As *ptx* influences mitotic spindle assembly, chromosome segregation, and cell division by inhibiting microtubule assembly [66], nuclear fragments can be a result of the action of intracellular *ptx* released from A-PHN. To evaluate drug delivery efficacy, the live and dead cells of the spheroid were stained after incubation of the spheroid with *ptx*@A-PHN under light exposure (532 nm laser, 3.5 W/cm^2^) or in the dark. As shown in Fig. 7c(i), the proportion of the red-colored dead cells in the spheroid was greater only in the light-illuminated group treated with *ptx*@A-PHN, indicating that the delivery efficacy was enhanced by the photothermally triggered release and rapid diffusion of *ptx* from A-PHN. To quantify this, the fluorescent signals of both live and dead cells were collected and the proportion of live/dead cells was calculated (Fig. 7c(ii)). In the case of the 30-min light exposure group using *ptx*@A-PHN, the ratio drastically decreased to 85.38% compared with that of the unexposed group. Collectively, drugs released from the light-responsive A-PHNs were observed to diffuse into the deeper region of the spheroid, killing more cells.

## Discussion

Light-responsive biomaterials are widely applicable in various biomedical fields including drug delivery systems, therapies, regenerative medicines, and optogenetics owing to their benefits such as easy controllability, high spatiotemporal resolution, and orthogonality to lots of biological and chemical processes [10, 11]. For controlled drug delivery, the light-responsive delivery carriers can improve therapeutic efficacy by on-demand regulation of drug kinetics in a spatiotemporal manner [24, 25]. Additionally, those carriers can directly use to treat the malignant tissues via photodynamic [28] or photothermal therapies [30] at a desired time and location. Although their outstanding therapeutic potentials, most of the light-responsive biomaterials require UV light for activation [26, 27], which might lead the phototoxicity in the body. Moreover, short-wavelength light undesirably interacts with the biological tissue, which might abnormally activate photochemical or photobiological processes.

In this study, we developed a facile synthetic method for the plasmonic hybrid nanogel (PHN) with adjustable light-responsive properties. The PHNs, basically composed of gold nanoparticles (GNPs) and the heat-sensitive nanogel, can be easily tuned for their physicochemical properties by changing the species of linker molecules during the synthetic process. For this purpose, we have applied 6-different biocompatible and functional linker molecules with different molecular weights; *N*,*N*′-methylene bis(acrylamide): a common crosslinker for polymerization of NIPAM [39], tryptophan: an amino acid with reducing power for gold ions [67, 68], sucrose and alginate: hydrophilic sugar molecules for the stabilization of GNPs [49, 51], poly(ethylene glycol) diacrylate: a linker molecule having acryl groups for NIPAM polymerization [69], and gelatin: a water-soluble protein for the capping of GNPs [70, 71]. Since the ligand-free GNPs were easily formed by free radicals generated from the photoinitiator [46, 47], they could pull the hydrophilic moieties of the polymeric network via weak interactive forces [48, 49]. To validate it, we observed the Au^0^ peaks which were shifted to a higher binding energy compared to the common metallic Au by using narrow scan XPS measurements (Additional file 1: Fig. S6). The peaks of O_1s_ and C_1s_ from polymeric networks indicated the interaction with GNPs. From these results, we can conclude that the GNPs are embedded in the polymeric network through weak interactive forces (e.g., Van der Waals, dipole–dipole, induced dipole, etc*.*) [46, 47, 49, 51, 52]. Additionally, we suggested that the improved structural stability of the hydrogels is also evidence of embedding GNPs in polymeric structures.

To adjust the hydrodynamic diameter, the integration degree of GNPs, and the morphologies of PHNs, we simply changed the linker molecules (Additional file 1: Fig. S7); hence we could effectively control the light-responsive properties including absorption and photothermal conversion rate of the PHNs. Especially, the alginate-linked PHN (i.e., A-PHN) exhibited about 4 times higher efficiency in the local heat generation compared to others. Since the optical properties of plasmonic nanostructures are affected not only by size and shape but also by the spatial arrangement [31, 32], this can be attributed to the densely clustered GNPs in the A-PHNs, of which increased optical cross-sectional area [53] induces higher photothermal conversion efficiency. Because our synthetic strategy is very simple, the aforementioned stereotypes could be modulated through the diversity of linker molecules which introduces a functional moiety like the Au–S conjugation [41], and/or post-synthetic modification [72]. For example, we could incorporate additional metallic nanoparticles into the PHN by chemical attachment or overgrowth methods [73, 74]. Thus, it is expected that absorption bands of the PHNs can be tunable to the NIR region (i.e., for better bio-orthogonality).

For controlled release as well as the endosomal escapes, we hypothesized that A-PHNs internalized in a cell generate the local heat which leads to their structural changes under light illumination. Owing to the difficulty in directly monitoring the photothermally-induced heat from the PHN, we indirectly confirmed it via observation of heat shock proteins (HSPs) expression. The heat shock response mechanism is essential to sustain cellular homeostasis and is highly conserved in all living organisms for adaptation to stress; hence it is obvious that the cytosolic HSPs were overexpressed under stress conditions [75, 76]. Among the HSP family, HSP90 participates in a molecular complex in the late stage since they do not act in nascent protein folding, unlike HSP70 [77]. Thus, we chose HSP90 as an indicator to prove the photothermal effects of PHNs inside the cell. As expected, the upregulation of HSP90 was observed with increasing the light exposure time. However, the mechanism of stress adaptation is very complex and needs to be considered with other biochemical processes to fully understand. To supplement our findings, we additionally performed the heat transfer simulation, which can prove the PHNs inside the vesicle could generate sufficient temperature for recruiting the HSPs [60, 78] as well as endosomal modification (i.e., vesicle rupture) [57–59].

Lastly, we have evaluated enhanced drug delivery efficacy of A-PHNs through deeper penetration by using the multicellular spheroids composed of melanoma cells and fibroblasts to mimic in vivo cellular microenvironment. Although multicellular spheroids have been widely applied in drug delivery fields owing to their high similarity to the tumor models [65, 79], there are still limitations to fully reflecting the preclinical/clinical models for developing translational medicine. For an in-depth understanding of the physiological/biochemical effect of A-PHNs, further studies using in vivo systems such as animal models are required.

## Conclusions

We suggested PHNs for spatiotemporally controllable delivery and pinpoint vesicular rupture through photothermally driven conformational changes. The PHNs were readily synthesized through the radical polymerization-based self-integration method in which GNPs were spontaneously embedded into the heat-sensitive nanogel (PNIPAM) and linker molecule during just 10 min reaction. The wave optics simulation revealed that the heat generation levels varied based on the densities of the GNPs and the sizes of the PHNs. After optimization, the alginate-linked PHNs (i.e., A-PHNs) exhibited the highest integration density for GNPs and a twofold elevated heat generation compared with that of the other candidates. The light-induced transient conformational changes of PNIPAM in A-PHNs permitted spatiotemporally controlled drug release for on-demand drug delivery into the cell. The photothermally boosted endosomal escape from the vesicular A-PHN facilitates intracellular delivery to the desired site. Finally, enhanced drug delivery efficiency was validated using 3D multicellular spheroids. We believe that the proposed method for synthesizing light- and heat-responsive hybrid nanocarriers and in-depth characterization of the drug release mechanism at the designated site provide a promising strategy for spatiotemporally controlled drug delivery.

## Methods

### Materials

*N*-Isopropyl acrylamide (> 97%, NIPAM), *N*,*N*′-methylene bis(acrylamide) (MBA), gold chloride trihydrate (HAuCl_4_), 2-hydroxy-2-methylpropiophenone (Darocur® 1173), tryptophan, sucrose, poly(ethylene glycol)-diacrylate (PEG-DA), alginic acid, gelatin, calcium chloride, paclitaxel, rhodamine 6G, and doxorubicin were purchased from Sigma-Aldrich (MO, USA). Ethanol (99.9%) and methanol (99.9%) were purchased from Samchun Chemical Co. (Seoul, South Korea). Live/dead cell imaging kits, Alexa Fluor® 488-phalloidin, Hoechst 33256, and LysoTracker™ Deep Red were purchased from Thermo Fisher Scientific (MA, USA). A polydimethylsiloxane (PDMS) elastomer kit (Sylgard 184) was purchased from Dow Corning (MI, USA). Anti-HSP90 antibody (D7a, ab59459) and goat anti-mouse IgG H&L (Alexa Fluor™ 488, ab150113) antibodies were purchased from Abcam (Cambridge, UK).

### Synthesis of plasmonic hybrid nanogels (PHNs)

A one-pot synthesis method for PHNs has been devised, in which plasmonic gold nanoparticles (i.e., GNPs) are spontaneously embedded in the process of polymerizing thermosensitive NIPAM-based nanogels. Before synthesis, all chemicals were dissolved in DI water and aspirated under N_2_ gas. First, 1% (w/v) NIPAM was mixed with 0.1% (w/v) linker molecules. A metal ion solution (10 mM HAuCl_4_) was added to the mixture in a 1:9 volume ratio. Then, 30 µL of Darocur® 1174 (10% v/v ethanol) was added to 1 mL of the reaction mixture and irradiated with UV light at 365 nm for 10 min. Subsequently, an equal volume of DI water was added to terminate the radical reaction. Finally, pure PHNs were separated through centrifugation at 4000 rpm for 20 min.

### Measurements of hydrodynamic diameter and zeta potential of the PHNs

An ELS-Z2000 light-scattering spectrophotometer (Otsuka Electronics, Japan) was used to analyze the hydrodynamic diameter and zeta potential. The polydispersity index was calculated as the square of the standard deviation divided by the square of the mean value for each sample (n = 3). All results were obtained in triplicate from individual samples. In the temperature-dependent experiment, the solution temperature ranged from 25 to 50 °C at 5 °C intervals.

### X-ray photoelectron spectroscopy (XPS)

XPS analyses were carried out with a Nexsa G2 spectrometer (ThermoFisher, USA) using a low-power Al K_α_ X-ray source. Spectra were obtained using an analysis area of approximately 400 × 600 µm. The spectra were measured with a 1 eV step size and 200 eV pass energy for survey resolution spectra, and 0.1 eV and 50 eV for narrow spectra, and were charge-corrected to the adventitious C_1s_ spectral components (C–C) with binding energy set to 284.8 eV.

### Wave optics computational simulation

Computational simulations were performed using COMSOL Multiphysics 5.2 (Comsol, Inc., USA). To calculate the photothermal heat source of the PHNs, a wave optics simulation was performed, and the absorption power (Q), was calculated for a single PHN. A plane TE-polarized electromagnetic wave with a wavelength range of 460–790 nm was applied. A heat transfer simulation was performed to obtain the temperature change inside the endocytic vesicle using a heat source. In the heat transfer simulation domain, the top and bottom surfaces were assumed to be under convection conditions with water.

### Raman spectroscopy

To measure the fingerprint spectra of the PHNs, a micro-Raman system was utilized, which was combined with a spectrophotometer (SR-303i, Andor Technology, UK) and an integral Olympus BX51 microscope with a 50× objective lens. The laser sources were a 532-nm laser (MSL-III-532, CNI Technology Co., China) and a 785-nm laser module (I0785SR0100B, Innovative Photonics Solution, USA). All Raman spectra were obtained with a 1 s exposure time (5 accumulations).

### High-resolution nuclear magnetic resonance (NMR) spectroscopy

NMR spectroscopy (Bruker AVANCE 600, Germany) was performed at various temperatures. The lyophilized samples were resuspended in D_2_O (1 mg/mL). The ^1^H-NMR spectrum of the sample was plotted using Brunker TopSpin® 4.0 software. Relative integral values were obtained by dividing the relative peak intensity of the secondary amine protons (denoted as *b*) by that of the benzophenone protons (denoted as *a*).

### Photothermal conversion of the PHNs

The photothermal properties of the PHNs were obtained by measuring the temperature change of the sample powders and solutions (1 mg/mL) under the irradiation of a 532-nm laser (3.5 W/cm^2^) and an LED (0.8 W/cm^2^, Futuregreen, Korea). The temperature was recorded using a fixed-mounted thermal imaging camera (FLIR A35sc, USA). Sample solutions (200 μL) in DI water were placed in front of the laser source or LED for 10 min to measure the photothermal conversion efficiency. After the light was turned off, the natural cooling period was monitored for 10 min. The temperature change was recorded every 5 s with an accuracy of 0.1 °C. The calculation of the photothermal conversion efficiency (*η*) is described in the supporting information.

### Fluorescent microscopy imaging

All fluorescent staining images were obtained by following standard protocols. Fluorescence images were acquired using a 20× objective lens on a fluorescence microscope equipped with an inverted microscope (DMi8, Leica, Germany). Phase-contrast and fluorescence images were processed using LAS X software. Confocal images were acquired using a confocal laser scanning microscope (LSM 800 with Airyscan, Carl Zeiss, Germany). Images were obtained using a water-immersed 20× and 40× objective lens. The obtained images were processed using the ZEN software. The primary antibody (D7a) was diluted to 0.1% in phosphate-buffered saline (PBS) and 0.1% tween® 20, and the secondary antibody was diluted to 0.1%. Colocalization images were obtained using the JACoP plugin using ImageJ software.

### MTT assay

A375P cells were seeded at a density of 100,000 cells/well in a 96-well plate containing 200 µL medium per well and incubated overnight. The medium was replaced with 200 µL of various sample solutions in PBS (pH 7.5). An LED was placed above the plate in the light illumination group for 30 min at RT. After the treatment, the samples were discarded and washed thrice. Then, a new medium was added to the cells and incubated overnight. The MTT-containing medium was then replaced with the cells and incubated for 3 h. To dissolve the tetrazolium, 200 µL dimethyl sulfoxide was added. Finally, the absorbance was measured at 550 nm and the background was measured at 650 nm using a microplate spectrophotometer (SpectraMax iD3, Molecular Devices, LLC., USA).

### Preparation of spheroids

Spheroids were formed on the PDMS-based substrate. The well structures, which were prepared by a previously reported elastomeric well for spheroid culture [80], were pre-treated with 3% BSA solution for 6 h to prevent cellular attachment at the bottom. The wells were then rinsed with PBS. A375P/HDF cells (2.5 × 10^5^ and 2.0 × 10^5^ cells/mL, respectively) were seeded into the wells directly and incubated for 5 days to form spheroids with a diameter over 150 µm.

### Statistical analysis

All experiments were performed at least in triplicate. The data are expressed as the mean ± standard deviation. The results were analyzed using a one-way analysis of variance (ANOVA) with the aid of OriginPro 8.0 software followed by Tukey’s test. Statistical significance (*) was set at p < 0.05.

## Supplementary Information

**Additional file 1: Text S1.** Calculation of the photothermal conversion efficiency. **Table S1.** Name and the molecular weight of the used linker molecules and nomenclatures of the PHNs synthesized with the linker molecules. **Fig S1.** Optimization of photoinitiator concentration for obtaining homogenous size distribution of GNPs. **Fig S2**. Hydrodynamic diameter distribution of the M-PHNs according to the reaction time between 1 and 15 min. **Fig S3.** Hydrodynamic diameters of PNIPAM nanogels without GNPs according to the reaction time. **Fig S4.** Absorbance spectra of colloidal GNPs and M-PHNs. Insets display the colors of the colloidal solutions. **Fig S5.** Monitoring of the thermal stability of M-PHNs and PHNs during 10 cycles of the heating/cooling procedure. **Fig S6.** Narrow scans of the XPS spectra focused on the selected elements of C, N, O, and Au. **Fig S7.** TEM images of the PHNs with different linker molecules (i.e., MBA (M-PHN), tryptophan (T-PHN), sucrose (S-PHN), PEG-da (P-PHN), alginate (A-PHN), and gelatin (G-PHN)). **Fig S8.** Optical properties and colloidal stabilities of the PHNs synthesized with different linker molecules. **Fig S9.** Molecular weights of alginate, PNIPAM, PNIPAM-alg, and A-PHN measured by SLS analysis. **Fig S10.** Solubility tests using the lyophilized A-PHN with various concentrations in water. **Fig S11.** Confirmation of the alginate incorporation in the A-PHN via calcium ion-mediated gelation method by adding 100 mM CaCl_2_. **Fig S12.** Energy-dispersive X-ray spectroscopy of A-PHN from Fig. 2g. **Fig S13.** Schematic image of the GNP structures used in the computation at different diameters of PHN. **Fig S14.** Linear relationship of -ln(θ) versus time obtained from the cooling period of the thermal curve in Fig. 3g. **Fig S15.** Light-responsive heat generation of dehydrated PHNs under light illumination. **Fig S16.** Monitoring of the solution temperature under a commercial LED. **Fig S17.** In situ Raman spectra of A-PHN gels under 785 nm laser illumination. **Fig S18.** Temperature-dependent ^1^H-NMR study of A-PHN dispersed in D_2_O. **Fig S19.** Standard curve of doxorubicin by absorbance at 480 nm. **Fig S20.** Drug release kinetics by different power densities of the LED. **Fig S21.** Monitoring the mean diameters of A-PHN by changing the buffer pH conditions. **Fig S22.** Monitoring the Raman spectra of released *dox* under temporally controlled light modulation. **Fig S23.** Colloidal stability of A-PHNs under different biological media. **Fig S24.** Biocompatibility tests using MTT assay to A375P. **Fig S25.** Images of Pearson’s colocalization coefficient (*k*) plots for *dox* versus *Hoechst* from Fig. 5e (i). **Fig S26.** Cellular internalization of A-PHNs into A375P cell. **Fig S27.** Computational simulation results of heat generation from vesicles including A-PHN. **Fig S28.** Observation of the damage to the cytoskeleton by A-PHNs under exposure to 532 nm laser at 3.5 W/cm^2^. **Fig S29.** Confocal fluorescent images of endocytic vesicles before and after laser illumination. **Fig S30.** Observation of nuclear fragments in A375P cells after treatment with *ptx*, A-PHN, and *ptx*@A-PHN (with light exposure), respectively.

## Data Availability

All data are available in the main text or the supplementary materials and are available from the corresponding authors upon reasonable request.

## Abbreviations

PHN: Plasmonic hybrid nanogel
A-PHN: Alginate-linked PHN
GNP: Gold nanoparticle
PNIPAM: Poly *N*-isopropyl(acrylamide)
LCST: Lower critical solution temperature
DOX: Doxorubicin
PTX: Paclitaxel
HSP: Heat shock protein
R6G: Rhodamine 6G
