# Supplementary material for: Spatiotemporally controlled drug delivery via photothermally driven conformational change of self-integrated plasmonic hybrid nanogels
Source: J Nanobiotechnology. 2023 Jun 14;21:191. doi: 10.1186/s12951-023-01935-x (PMC10265783; doi:10.1186/s12951-023-01935-x)
Supplement: Supplementary file 1 — Additional file 1: Text S1. Calculation of the photothermal conversion efficiency. Table S1. Name and the molecular weight of the used linker molecules and nomenclatures of the PHNs synthesized with the linker molecules. Fig S1. Optimization of photoinitiator concentration for obtaining homogenous size distribution of GNPs. Fig S2. Hydrodynamic diameter distribution of the M-PHNs according to the reaction time between 1 and 15 min. Fig S3. Hydrodynamic diameters of PNIPAM nanogels without GNPs according to the reaction time. Fig S4. Absorbance spectra of colloidal GNPs and M-PHNs. Insets display the colors of the colloidal solutions. Fig S5. Monitoring of the thermal stability of M-PHNs and PHNs during 10 cycles of the heating/cooling procedure. Fig S6. Narrow scans of the XPS spectra focused on the selected elements of C, N, O, and Au. Fig S7. TEM images of the PHNs with different linker molecules (i.e., MBA (M-PHN), tryptophan (T-PHN), sucrose (S-PHN), PEG-da (P-PHN), alginate (A-PHN), and gelatin (G-PHN)). Fig S8. Optical properties and colloidal stabilities of the PHNs synthesized with different linker molecules. Fig S9. Molecular weights of alginate, PNIPAM, PNIPAM-alg, and A-PHN measured by SLS analysis. Fig S10. Solubility tests using the lyophilized A-PHN with various concentrations in water. Fig S11. Confirmation of the alginate incorporation in the A-PHN via calcium ion-mediated gelation method by adding 100 mM CaCl2. Fig S12. Energy-dispersive X-ray spectroscopy of A-PHN from Fig. 2g. Fig S13. Schematic image of the GNP structures used in the computation at different diameters of PHN. Fig S14. Linear relationship of -ln(θ) versus time obtained from the cooling period of the thermal curve in Fig. 3g. Fig S15. Light-responsive heat generation of dehydrated PHNs under light illumination. Fig S16. Monitoring of the solution temperature under a commercial LED. Fig S17. In situ Raman spectra of A-PHN gels under 785 nm laser illumination. Fig S18. Temperature-de [file 12951_2023_1935_MOESM1_ESM.pdf]

Additional file 1

Spatiotemporally Controlled Drug Delivery *via* Photothermally Driven Conformational Change of Self-integrated Plasmonic Hybrid Nanogels

Seungki Lee[1], Subeen Kim[3], Doyun Kim[1], Jieun You[1], Ji Soo Kim[4], Hakchun Kim[1], Jungwon Park[4,5], Jihwan Song[3], and Inhee Choi[1,2]**

*Corresponding authors: inheechoi1@uos.ac.kr; jsong@hanbat.ac.kr

Text S1

Calculation of the photothermal conversion efficiency

The photothermal conversion efficiency (η) of the PHNs was calculated according to a previous report. The detailed method is as follows:

$$\eta = \frac{hs(T_{max} - T_{Sur}) - Q_{Dis}}{I(1-10^{-Abs})} \quad (1)$$

where h is the heat transfer coefficient, s is the surface area of the container.

To gain hs, a dimensionless parameter θ is introduced as followed:

$$\theta = \frac{T - T_{Sur}}{T_{max} - T_{Sur}} \quad (2)$$

And, a time constant (t) can be calculated by (3).

$$t = -\tau_s \ln(\theta) \quad (3)$$

According to **Fig. S14**, τ_s was determined and calculated to be 203.20 s.

$$hs = \frac{m_D C_D}{\tau_s} \quad (4)$$

Additionally, m was 0.2 g and C was found to be 4.2 J/g°C. Thus, according to (4), hs was calculated to be 6.20 mW/°C for A-PHN.

In our study, the maximum steady temperature (T_{max}) and the surrounding temperature (T_{Sur}) of A-PHN dispersion were measured at 36.2°C and 25.3°C, respectively. The value of (T_{Max}-T_{Sur}) was calculated to 10.9°C. In the case of laser power I, was 0.2 W. The absorbance of A-PHNs at 532 nm was 1.0. Q_{Dis} expresses the heat dissipated from the light absorbed by the container itself filled with pure water (15 mW).

Therefore, substituting the values of each parameter in (1), the 532 nm laser-mediated heat conversion efficiency (η) of the A-PHNs was calculated to be 15.69%.

Table S1. Name and the molecular weight of the used linker molecules and nomenclatures of the PHNs synthesized with the linker molecules.

PHNs	Linker molecules	M.W. (g/mol)
M-PHN	N,N`-methylene bis(acrylamide)	154.17
T-PHN	Tryptophan	204.23
S-PHN	Sucrose	342.3
P-PHN	Poly(ethylene glycol) diacrylate	575.5
A-PHN	Alginic acid	M_n: 58,900
G-PHN	Gelatin	50,000-100,000

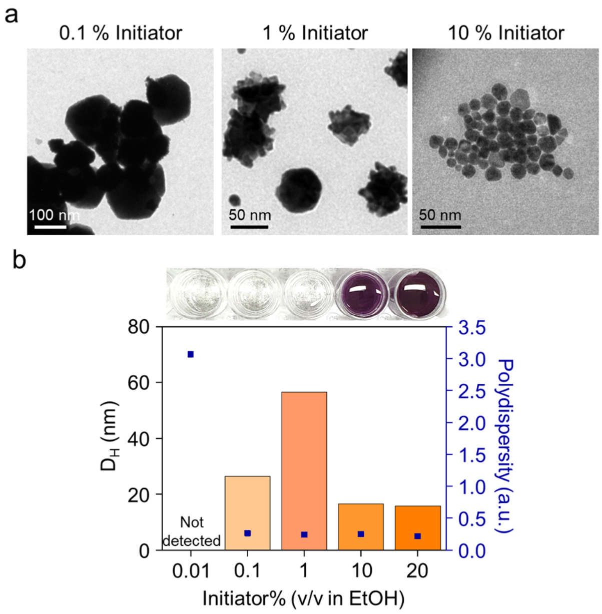

Fig. S1. Optimization of photoinitiator (PI) concentration for obtaining homogenous size distribution of gold nanoparticles (GNPs). (**a**) Transmission electron microscopy (TEM) images of GNPs according to the PI concentration. (**b**) Hydrodynamic diameters and polydispersity of the obtained GNPs. The photographs indicate the colloidal colors of GNPs formed through radical polymerization.

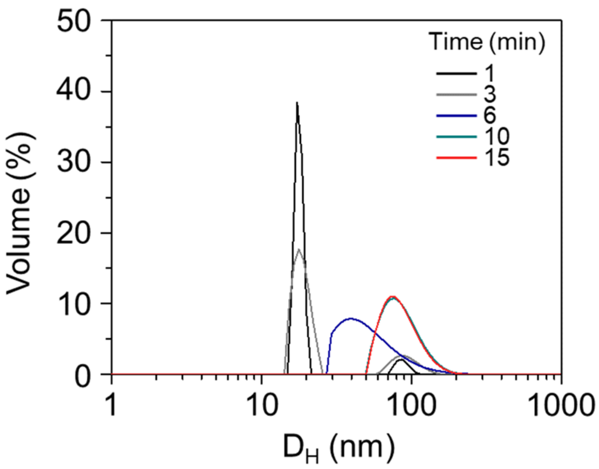

Fig. S2. Hydrodynamic diameter distribution of the *N, N'*-methylene bisacrylamide (MBA)-linked PHNs (M-PHNs) according to the reaction time between 1–15 min.

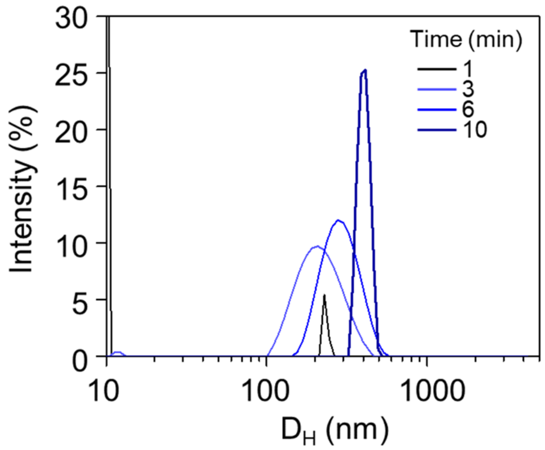

Fig. S3. Hydrodynamic diameters of poly (*N*-isopropyl acrylamide) (PNIPAM) nanogels without GNPs according to the reaction time.

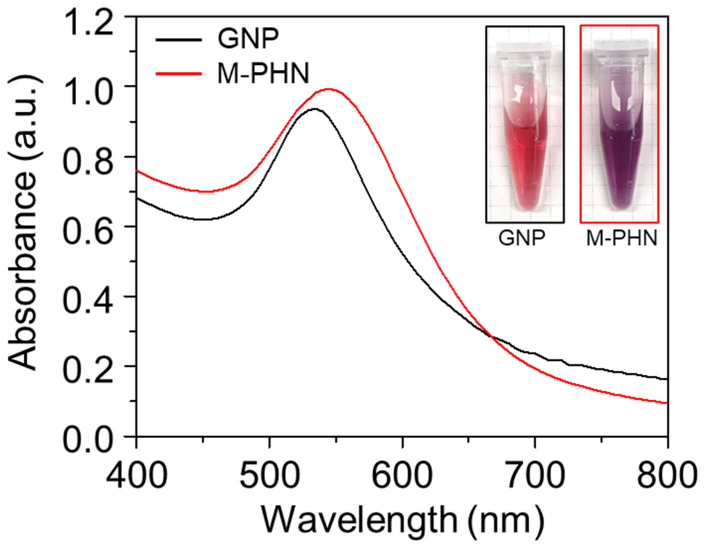

Fig. S4. Absorbance spectra of colloidal GNPs and M-PHNs. Insets display the colors of the colloidal dispersions.

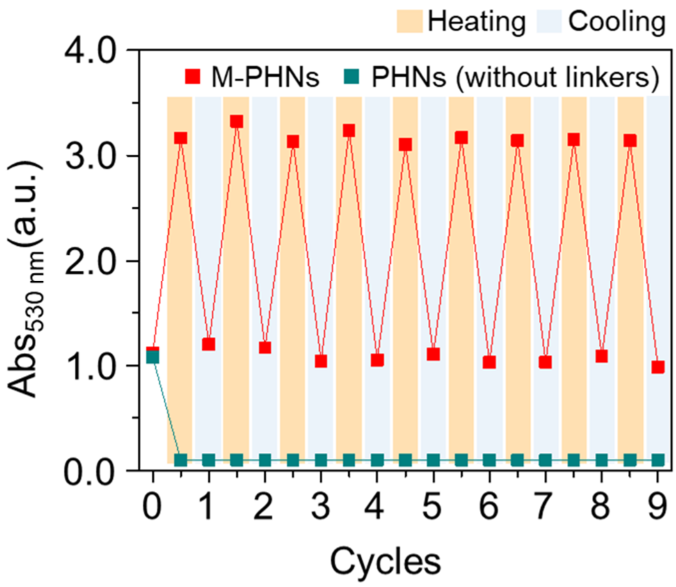

Fig. S5. Monitoring of the thermal stability of M-PHNs and PHNs during 10 cycles of the heating/cooling procedure.

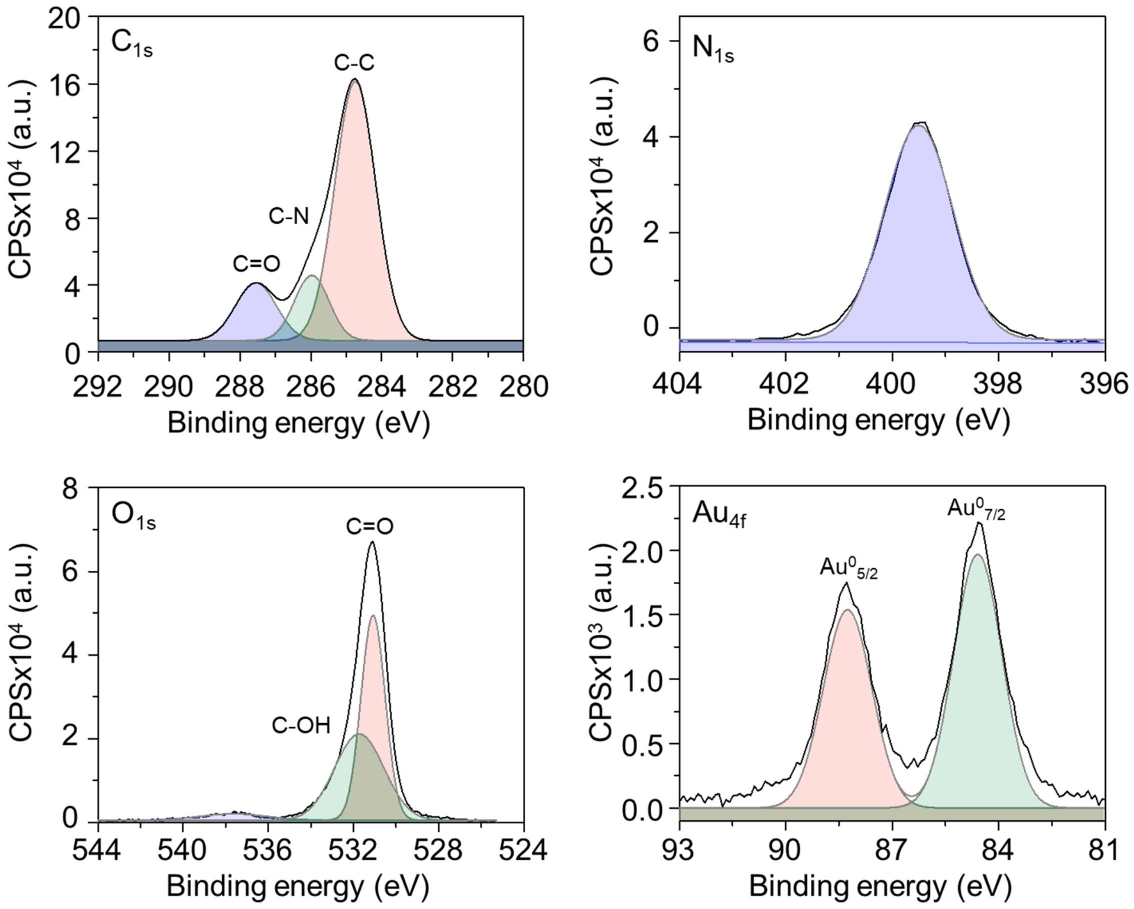

Fig. S6. Narrow scans of the XPS spectra focused on the selected elements of C, N, O, and Au.

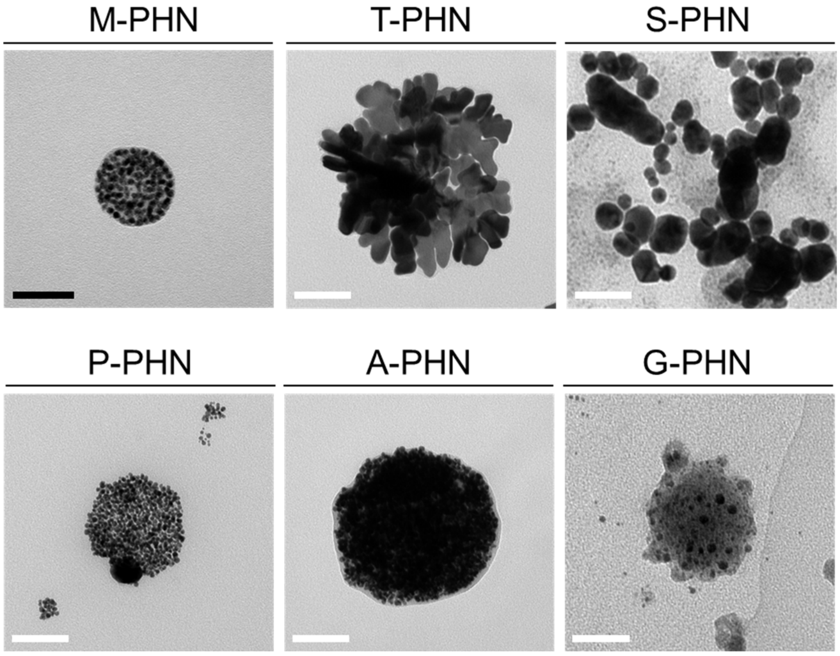

Fig. S7. TEM images of the PHNs with different linker molecules (*i.e.*, MBA (M-PHN), tryptophan (T-PHN), sucrose (S-PHN), PEG-da (P-PHN), alginate (A-PHN), and gelatin (G-PHN)). The Black scale bar indicates 50 nm and the white scale bars represent 100 nm.

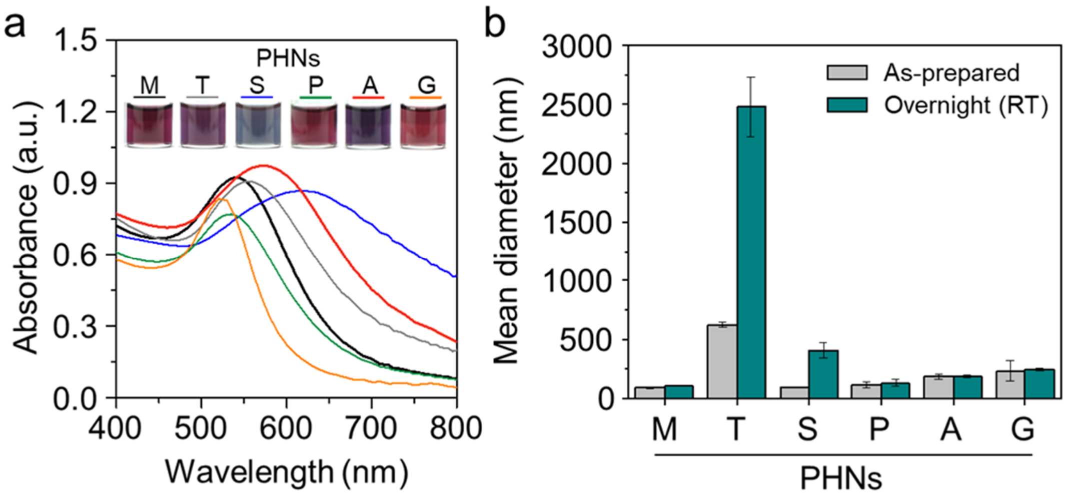

Fig. S8. Optical properties and colloidal stabilities of the PHNs synthesized with different linker molecules. (**a**) Absorbance spectra and photographs of the PHNs. (**b**) Mean diameters were measured from as-synthesized colloidal PHNs and the PHNs stored at RT (25°C) overnight.

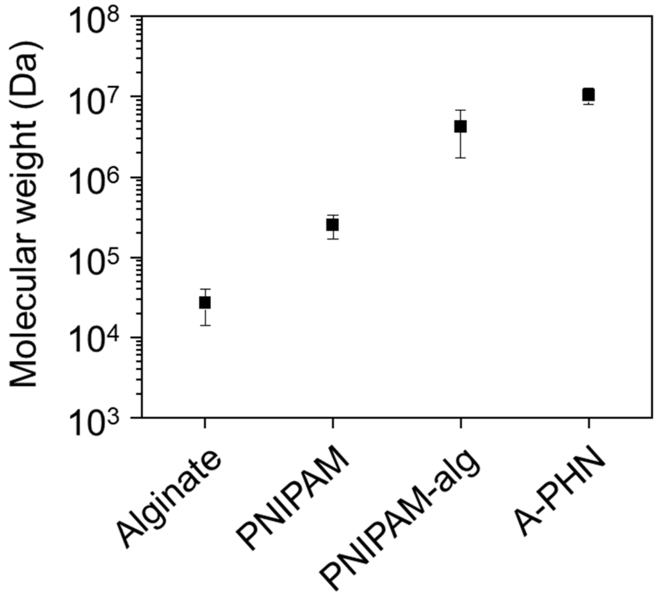

Fig. S9. Molecular weights of alginate, PNIPAM, PNIPAM-alg, and A-PHN measured by SLS analysis.

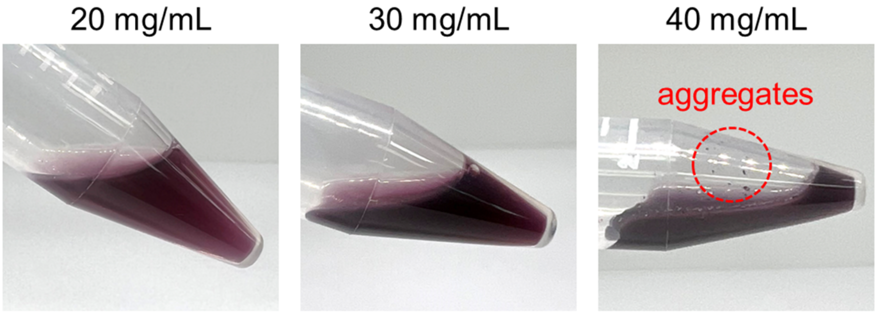

Fig. S10. Water solubility of the lyophilized A-PHN.

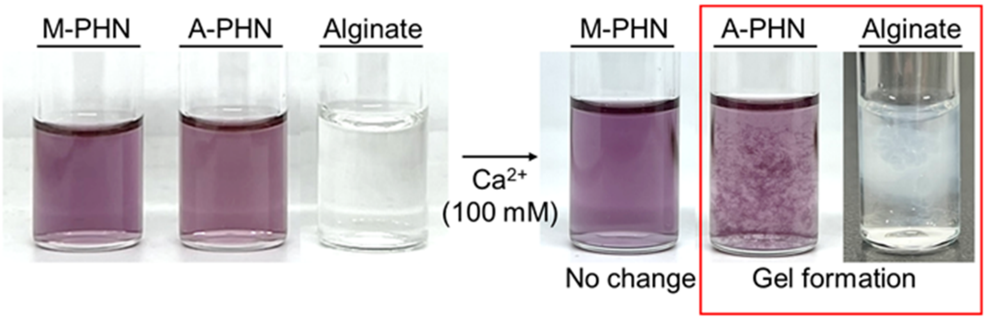

Fig. S11. Confirmation of the alginate incorporation in the A-PHN *via* calcium ion-mediated gelation method by adding 100 mM $CaCl_2$.

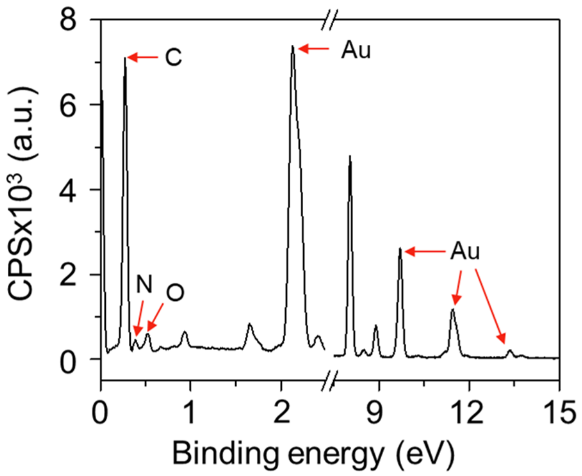

Fig. S12. Energy-dispersive X-ray spectroscopy of A-PHN from **Fig. 2g**.

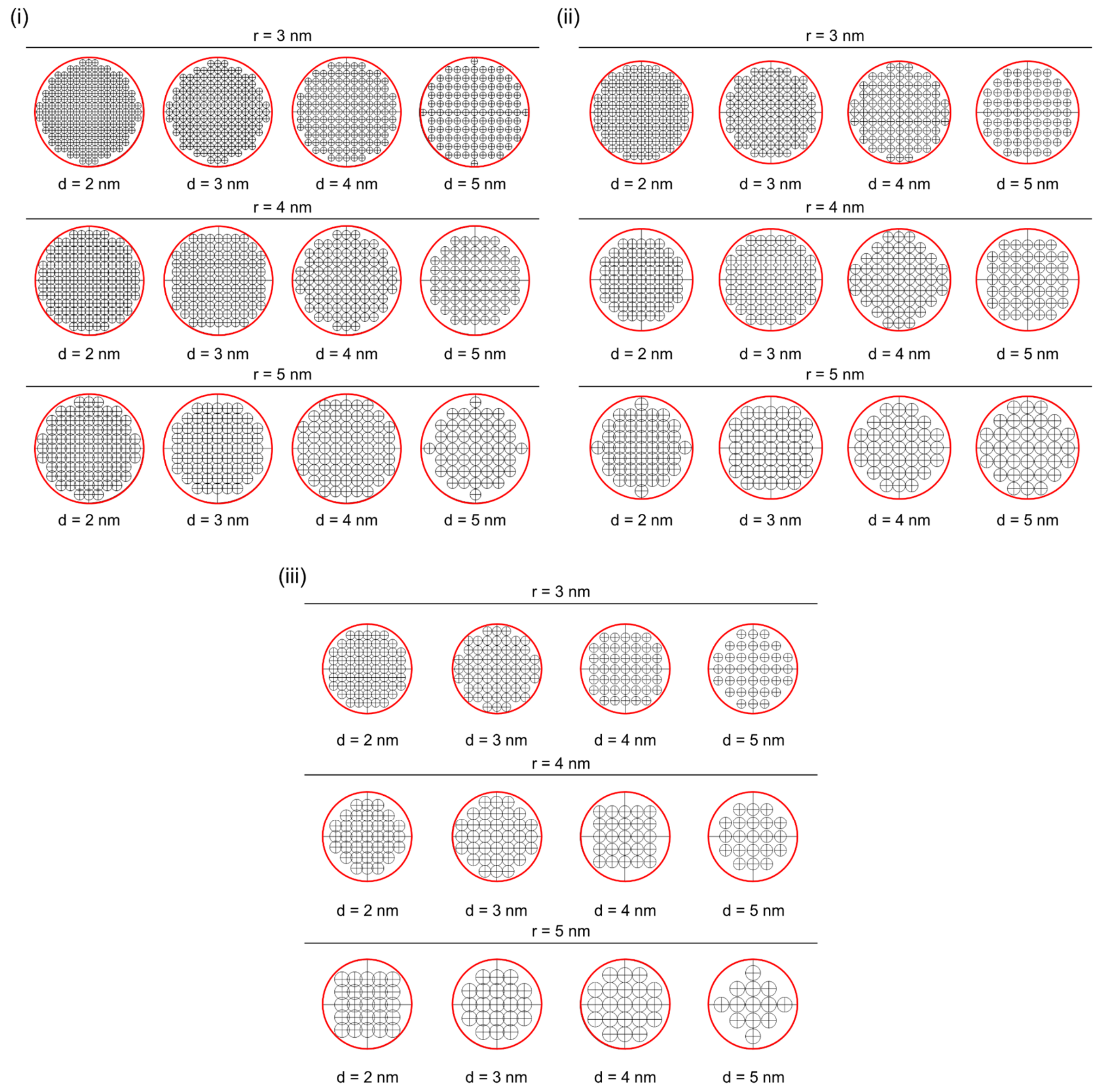

Fig. S13. Schematic image of the GNP structures used in the computation at different diameters of PHN (i) R=100 nm, (ii) R=80 nm, and (iii) R=60 nm, respectively. The red line indicates the boundary of the PHN.

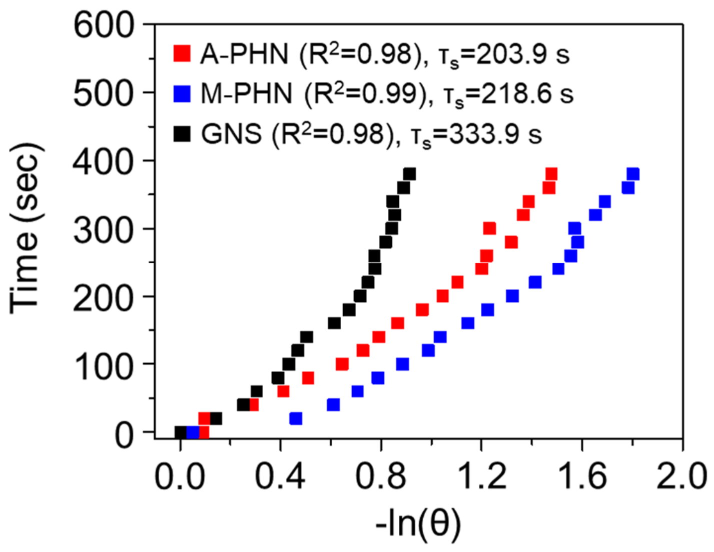

Fig. S14. Linear relationship of -ln(θ) versus time obtained from the cooling period of the thermal curve in **Fig. 3g**. A dimensionless driving force temperature, θ was obtained using the equation, $\theta = (T - T_{Sur})/(T_{Max}\text{-}T_{Sur})$, where T_{Sur} indicates the environmental temperature, and T_{max} represents the maximum steady temperature, respectively.

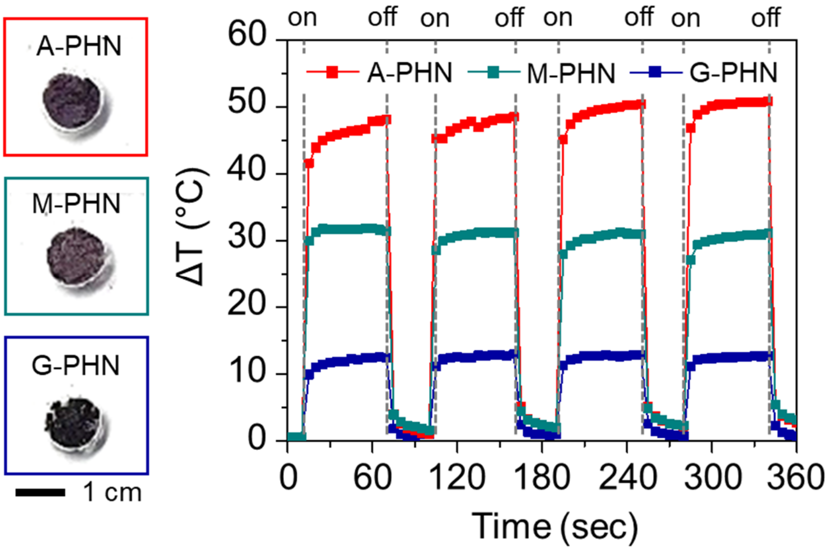

Fig. S15. Light-responsive heat generation of dehydrated PHNs under light illumination (532 nm, 0.8 W/cm^2). Gray dash lines indicate the time points on/off the laser.

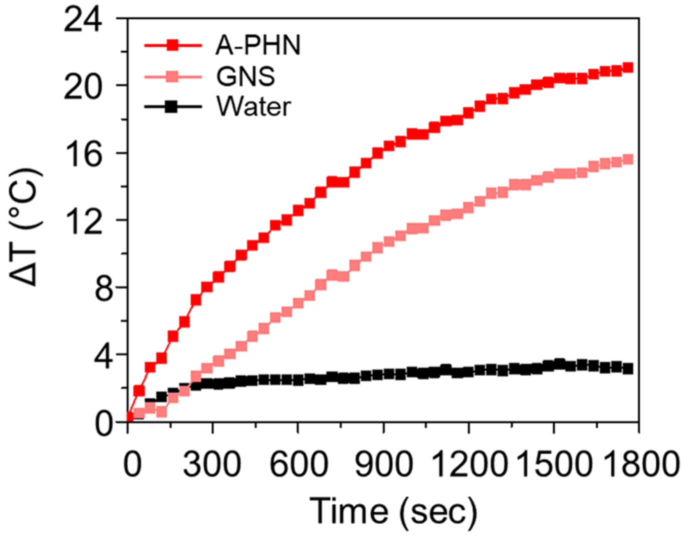

Fig. S16. Monitoring of the solution temperature under a commercial LED (0.8 W/cm^2).

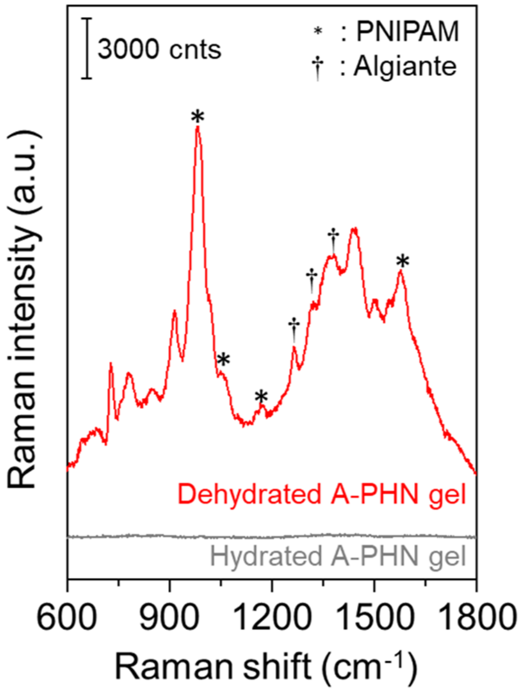

Fig. S17. In situ Raman spectra of A-PHN gels under 785-nm laser illumination (* indicate the hydrophobic moieties at PNIPAM nanogel, † represents the fingerprint peak from alginate). Each gel was prepared using Ca^{2+}-induced gelation, and a dehydrated state was obtained by heating the solution over 40℃.

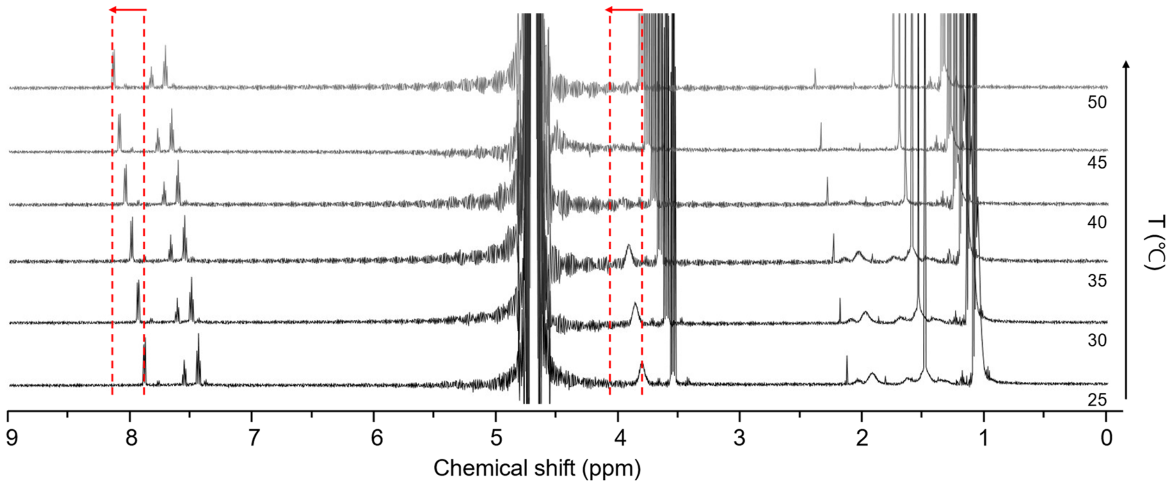

Fig. S18. Temperature-dependent ^{1}H-NMR (600 MHz) study of A-PHN dispersed in D_2O.

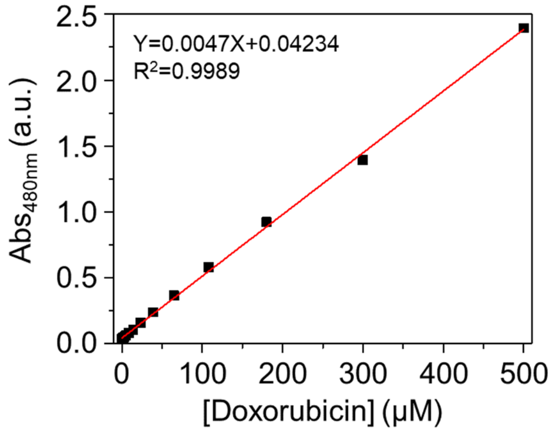

Fig. S19. Standard curve of doxorubicin by absorbance at 480 nm.

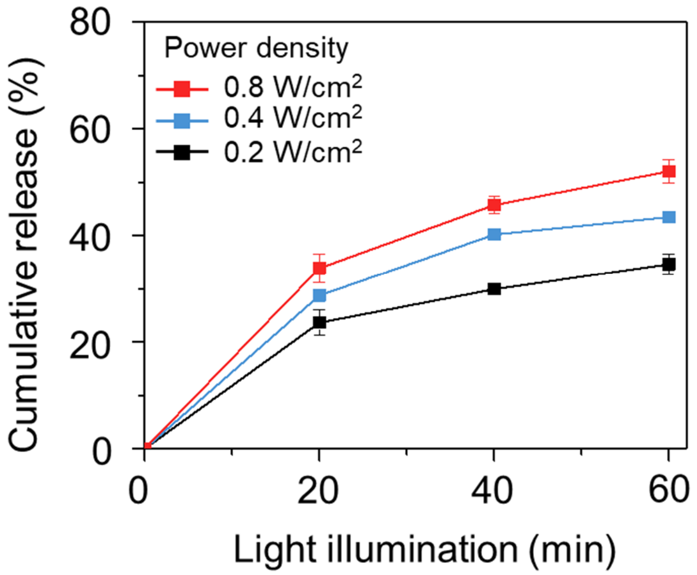

Fig. S20. Drug release kinetics by different power densities of the LED (n=3).

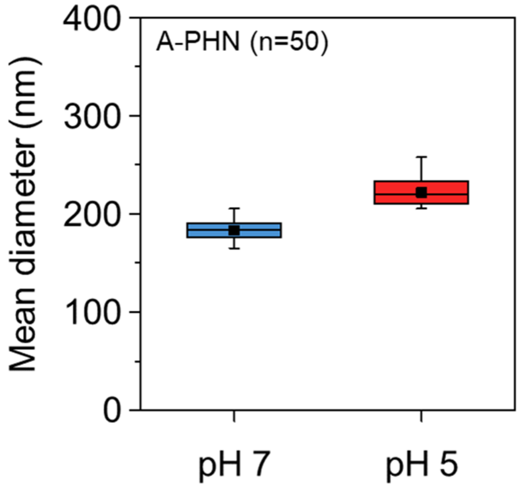

Fig. S21. Monitoring the mean diameters of A-PHN by changing the buffer pH conditions (n=50).

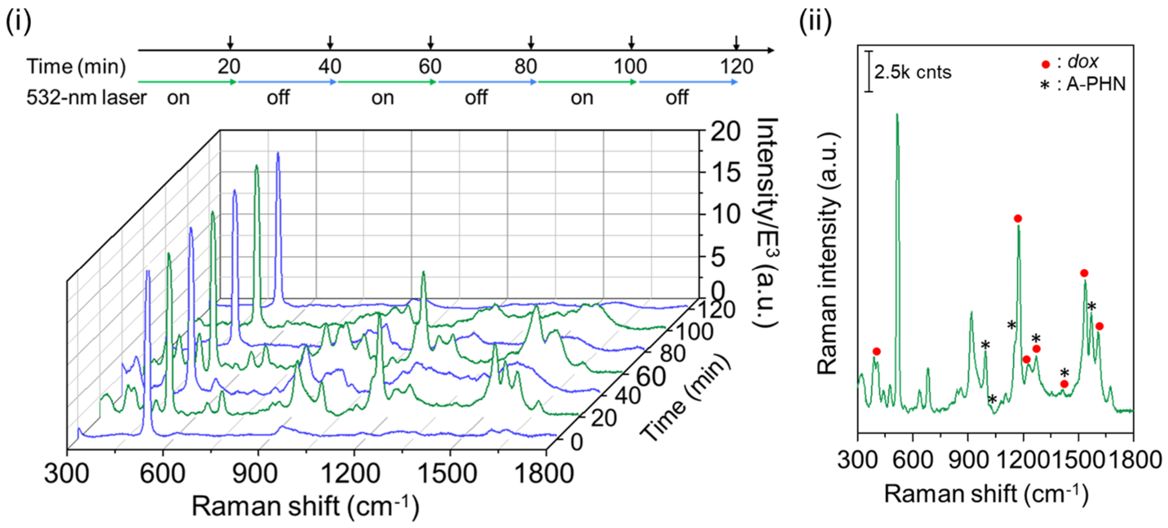

Fig. S22. Monitoring the Raman spectra of released *dox* under temporally controlled l ight modulation. (i) Time-coursed Raman spectra. (ii) Representative spectrum collected at every 20 min of 532 nm illumination. Raman spectra were obtained using the 785 nm laser with 1 s of exposure time (5 times accumulation each).

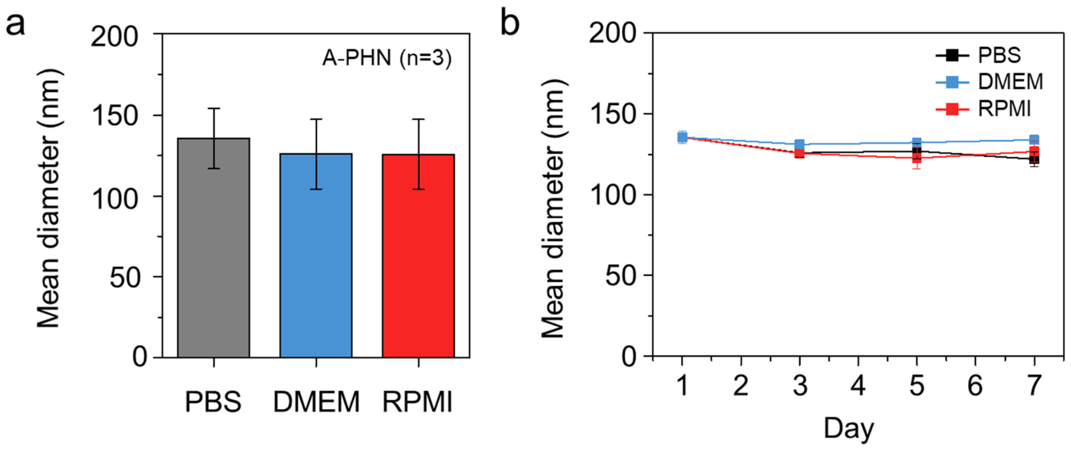

Fig. S23. Colloidal stability of A-PHNs. (a) Mean diameters of the A-PHNs under different biological media (n=3), (b) Monitoring of mean diameters of the A-PHNs for 7 days.

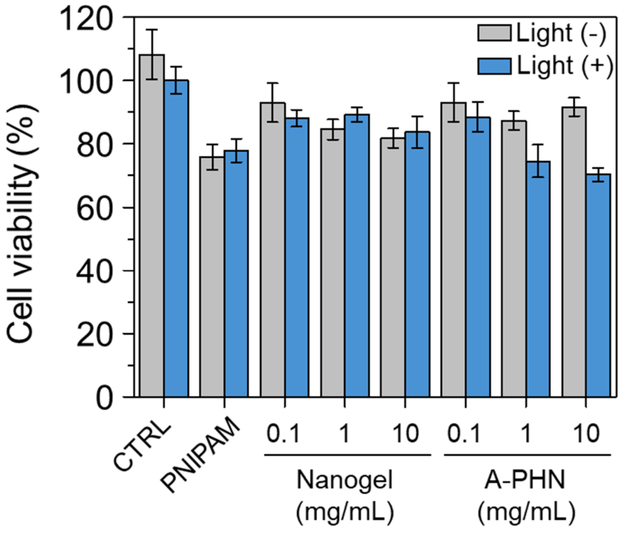

Fig. S24. Cell viability tests of A-PHNs and nanogels (n=5).

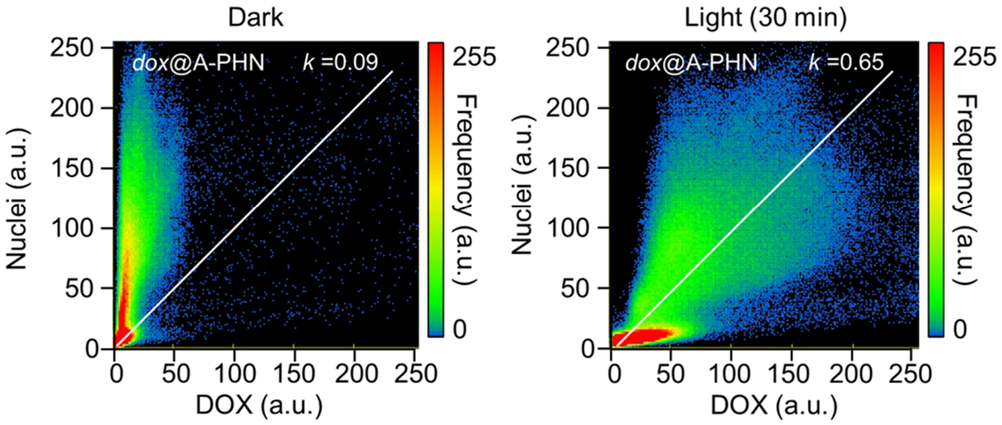

Fig. S25. Images of Pearson's colocalization coefficient (*k*) plots for *dox* versus *Hoechst* from **Fig. 5e (i)**.

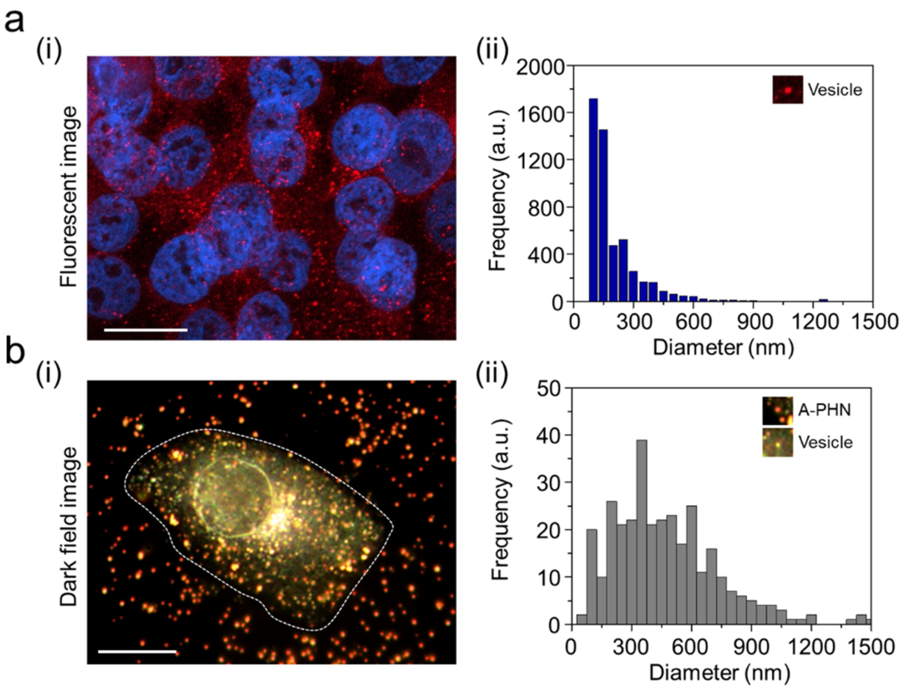

Fig. S26. Cellular internalization of A-PHNs into A375P cell. (**a**) Confocal microscopy analysis of endocytic vesicles. (i) Fluorescence image when stained with DND-99 (red) and *Hoechst 33258* (blue). The scale bar is 20 µm. (ii) Frequency plots extracted from red dots from the fluorescent images. (**b**) Dark-field scattering microscopy results. (i) Scattering image of the representative single cell including A-PHN (*i.e.*, orange scattering spots). The scale bar is 40 µm. (ii) Frequency plots from scattering spots. All distribution plots were obtained from ImageJ.

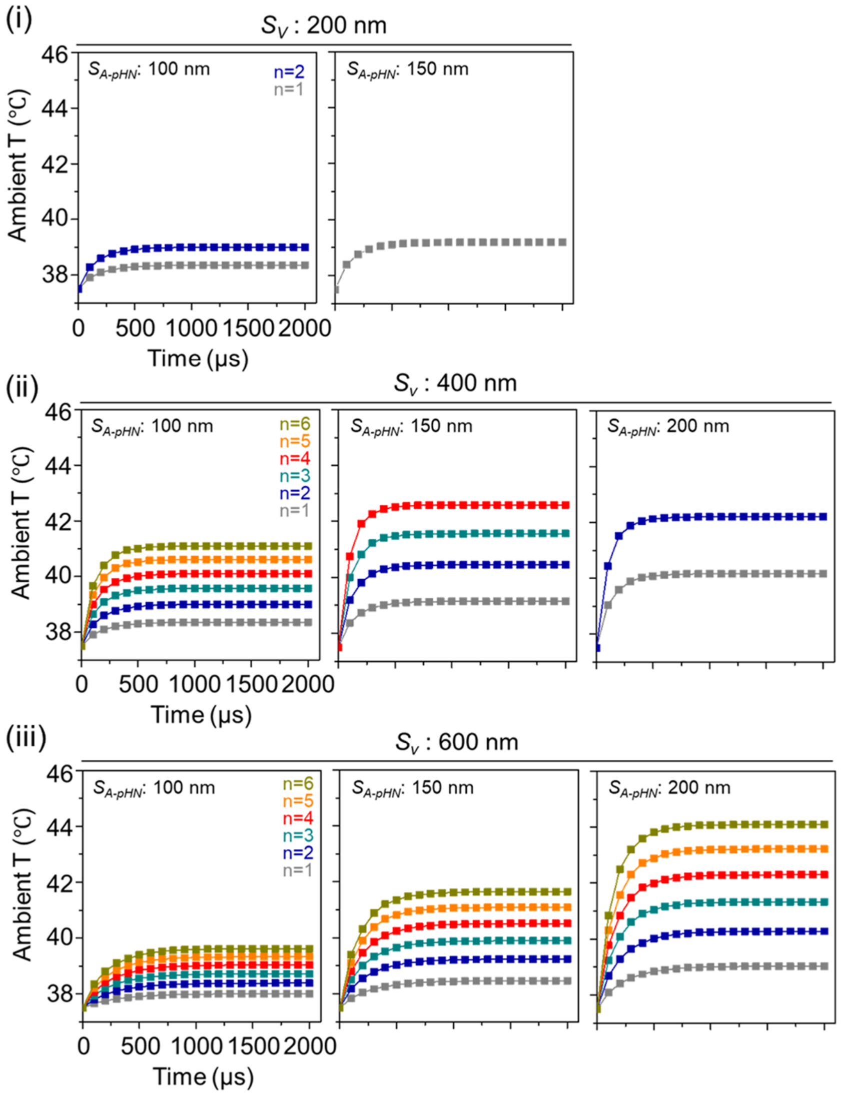

Fig. S27. Computational simulation results of heat generation from vesicles including A-PHN. Heat elevation profile using (i) S_v=200 nm, (ii) S_v=400 nm, and (iii) S_v=600 nm with different sizes ($S_{A\text{-}PHN}$) and numbers (n) of PHN.

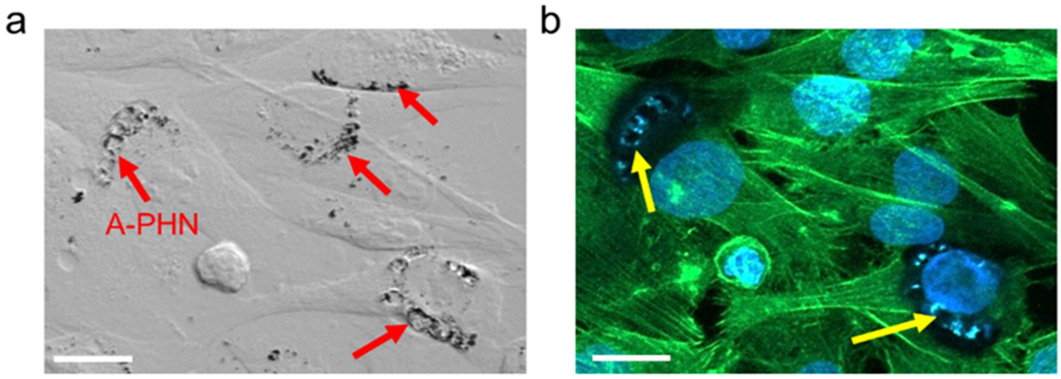

Fig. S28. Observation of the damage to the cytoskeleton by A-PHNs under exposure to 532 nm laser at 3.5 W/cm^2. Black dots indicate the A-PHN, the green color indicates F-actin, and blue indicates the nuclei. Scale bars are 20 µm. (**a**) Bright-field image after the laser illumination on the A-PHN. (**b**) Fluorescence image of the same area. Red arrows indicate A-PHN and yellow arrows indicate the damaged areas.

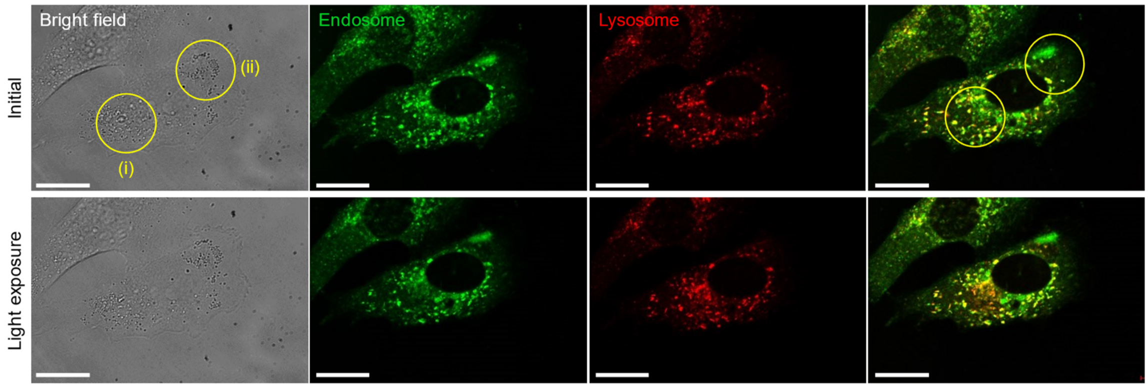

Fig. S29. Confocal fluorescent images of endocytic vesicles before and after laser illumination (Scale bar: 20 µm). Green (WGA-640R) and red (DND-99) spots indicate endosomes and lysosomes, respectively. (i) represents the late endosome region, and (ii) indicates the early endosome site. A 550 nm laser (3.5 W/cm^2) was utilized for light stimulation.

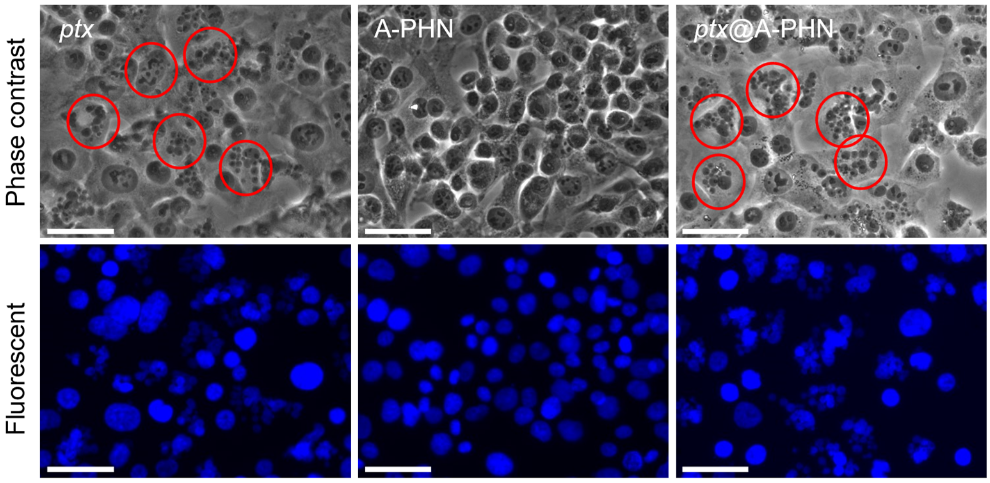

Fig. S30. Observation of nuclear fragments in A375P cells after treatment with *ptx*, A-PHN, and *ptx*@A-PHN (with light exposure), respectively. (Scale bar: 50 µm) Nuclei were stained with *Hoechst*. Red circles indicate the *ptx*-induced apoptotic nuclear fragmentations.
